# Discovery of a Splicing Regulator Required for Cell Cycle Progression

**DOI:** 10.1371/journal.pgen.1003305

**Published:** 2013-02-21

**Authors:** Elena S. Suvorova, Matthew Croken, Stella Kratzer, Li-Min Ting, Magnolia Conde de Felipe, Bharath Balu, Meng L. Markillie, Louis M. Weiss, Kami Kim, Michael W. White

**Affiliations:** 1 Departments of Molecular Medicine and Global Health and the Florida Center for Drug Discovery and Innovation, University of South Florida, Tampa, Florida, United States of America; 2 Department of Medicine, Albert Einstein College of Medicine, Bronx, New York, United States of America; 3 Department of Microbiology and Immunology, Albert Einstein College of Medicine, Bronx, New York, United States of America; 4 Department of Animal Pathology, University of Las Palmas de Gran Canaria, Arucas, Las Palmas de Gran Canaria, Spain; 5 Tropical Disease Research Program, Center for Infectious Disease and Biodefense Research, SRI International, Harrisonburg, Virginia, United States of America; 6 Fundamental and Computational Sciences, Pacific Northwest National Laboratory, Richland, Washington, United States of America; Centre National de la Recherche Scientifique, France

## Abstract

In the G1 phase of the cell division cycle, eukaryotic cells prepare many of the resources necessary for a new round of growth including renewal of the transcriptional and protein synthetic capacities and building the machinery for chromosome replication. The function of G1 has an early evolutionary origin and is preserved in single and multicellular organisms, although the regulatory mechanisms conducting G1 specific functions are only understood in a few model eukaryotes. Here we describe a new G1 mutant from an ancient family of apicomplexan protozoans. *Toxoplasma gondii* temperature-sensitive mutant 12-109C6 conditionally arrests in the G1 phase due to a single point mutation in a novel protein containing a single RNA-recognition-motif (TgRRM1). The resulting tyrosine to asparagine amino acid change in TgRRM1 causes severe temperature instability that generates an effective null phenotype for this protein when the mutant is shifted to the restrictive temperature. Orthologs of TgRRM1 are widely conserved in diverse eukaryote lineages, and the human counterpart (RBM42) can functionally replace the missing *Toxoplasma* factor. Transcriptome studies demonstrate that gene expression is downregulated in the mutant at the restrictive temperature due to a severe defect in splicing that affects both cell cycle and constitutively expressed mRNAs. The interaction of TgRRM1 with factors of the tri-SNP complex (U4/U6 & U5 snRNPs) indicate this factor may be required to assemble an active spliceosome. Thus, the TgRRM1 family of proteins is an unrecognized and evolutionarily conserved class of splicing regulators. This study demonstrates investigations into diverse unicellular eukaryotes, like the Apicomplexa, have the potential to yield new insights into important mechanisms conserved across modern eukaryotic kingdoms.

## Author Summary

The study of eukaryotic cell division has overwhelmingly focused on cells from two branches of evolution, fungal and metazoan, with more distant eukaryotes rarely studied. One exception is apicomplexan pathogens where in the last two decades development of genetic models has been rapid. While not a perfect solution to fill the missing evolutionary diversity, Apicomplexans represent one of the oldest eukaryotic lineages possibly pre-dating the divergence of plant and animal kingdoms. A key to uncovering novel and conserved cell cycle mechanisms in these protists was the development of forward genetic approaches that permit unbiased discovery of essential growth factors. The apicomplexan, *Toxoplasma* has provided the best resource so far with ∼60,000 chemical mutants yielding a collection of 165 temperature-sensitive isolates that arrest in all phases of the parasite cell cycle. Efforts to identify the defective genes in this model are providing insights into the regulatory factors possibly active in the original eukaryote cell cycle, like the mRNA splicing factor discovered in this study.

## Introduction

Protozoans belonging to the Apicomplexa were recently combined with two other groups the ciliates and dinoflagellates to form a new monophyletic group called the Alveolata [1]. Unicellular Alveolates arose early in the evolution of eukaryotes, and while the placement of this branch before or after the separation of the plant and animal kingdoms is controversial [2], [3], there is no disputing this large collection of protists has an ancient origin. By some estimates the age of Alveolate divergence exceeds a billion years [2]. Modern Alveolates are found throughout the world where they have successfully adopted free and/or parasitic life styles. Many important species in the Apicomplexan subgroup are human pathogens including five members of the *Plasmodium* genus that cause malaria. *Plasmodium falciparum* is responsible for nearly a million deaths annually with this disease concentrated in central Africa affecting many children under 5 years old [4].

Exploring the cell biology of these ancient eukaryotes reveals novel features have evolved to ensure cell growth and proliferation of Alveolates in diverse environments. Nothing illustrates this adaption better than the elaborate counting mechanisms that enable Dinoflagellates to switch their cell cycle from binary to multinuclear in different nutrient conditions [5] or have allowed Apicomplexans to reproduce at scales (2 to >25,000 divisions) matched to the choice of host cell [6]. To ensure transmission to the next host, the Apicomplexa devote considerable resources to construct their invasion apparatus at the right time in mitosis, which provides another remarkable example of novel processes integrated into the classic eukaryotic mitosis. Given the peculiar features documented it is tempting to speculate that control of Alveolate replication is different from other eukaryotes. When key checkpoint proteins are not detected in genome sequence [6] this conclusion seems at least partially correct. Yet, most Alveolate cell cycles have some transitions that are similar to division cycles of eukaryotes from multicellular kingdoms. Interphases comprised of conventional G1 and S phases (G2 maybe less conserved) are common [5], [6], [7] and cyclin-CDK factors present in Alveolate genomes [6], [8] are assumed to check the fidelity of cell cycle transitions as they do in other eukaryotes [9]. Segregation of nuclear chromosomes in Alveolates requires microtubule organizing structures and the timing of mitosis likely utilizes some version of the anaphase promoting complex whose components are also found in Alveolate genome sequence [6]. These distinct cell cycle views of old and new suggest we have much to learn about the mechanisms working in these protozoa to achieve their diverse replication schemes. Recent reflections on the cell cycles of fungi and animals [9], indicates we should expect regulatory factor divergence even where protozoan cell cycles appear to have preserved the same network topology working to control multicellular eukaryote division. Not surprising, all expected levels of protein conservation from pan-eukaryote to species-specific growth factors are emerging from unbiased genetic screens now successfully developed for the study of *Toxoplasma gondii* cell division (Suvorova and White, unpublished) [8], [10], [11].

Model organisms among these ancient protozoa are valuable because they offer insight into the flexibility possible in the eukaryotic cell cycle and at the same time will help define core cell cycle mechanisms preserved since the first eukaryote. *Toxoplasma* has emerged as a principal genetic system from the Apicomplexa with particular strengths in the study of cell cycle mechanisms [8]. The binary replication of the *Toxoplasma* tachyzoite is relatively simple composed of major G1, S, and mitotic phases [12]. Internal budding that is a hallmark of the apicomplexan division begins late in the tachyzoite S phase and spans the classical mitotic events necessary for chromosome segregation ultimately resulting in two infectious daughter parasites [6], [7]. There is a rough demarcation of old and new cell cycle processes in the two halves of the tachyzoite division cycle that was borne out in the *Toxoplasma*, and also the *Plasmodium falciparum*, cell cycle transcriptomes [13], [14] suggesting this is a general expression scheme among the Apicomplexa. The cell cycle transcriptome of these protozoa is characterized by a serial progression of cyclical mRNAs with canonical growth factors reaching a maximum in G1 followed by peak expression of dozens of specialized genes needed for building the invasion apparatus in S and mitotic phases [13]. How this transcriptional cascade is regulated is unknown nor do we understand the intersection between cell cycle gene expression and the checkpoint mechanisms orchestrated by cyclin-CDK factors present in these protozoa.

Here we describe a cell cycle mutant in *Toxoplasma* that is defective in a fundamental eukaryotic cell cycle function. This defect results in a rapid growth arrest in the G1 phase, which is not recoverable when cells are shifted back to a permissive temperature. The novel RNA binding protein discovered by genetic rescue is conserved in many modern eukaryotes such that the human ortholog can fully replace the function of the *Toxoplasma* mutant protein. We show that this new family of proteins is required for gene expression where it promotes proper mRNA splicing.

## Results

### Isolation of a cell cycle mutant whose growth is conditional for temperature

**Figure 1:**
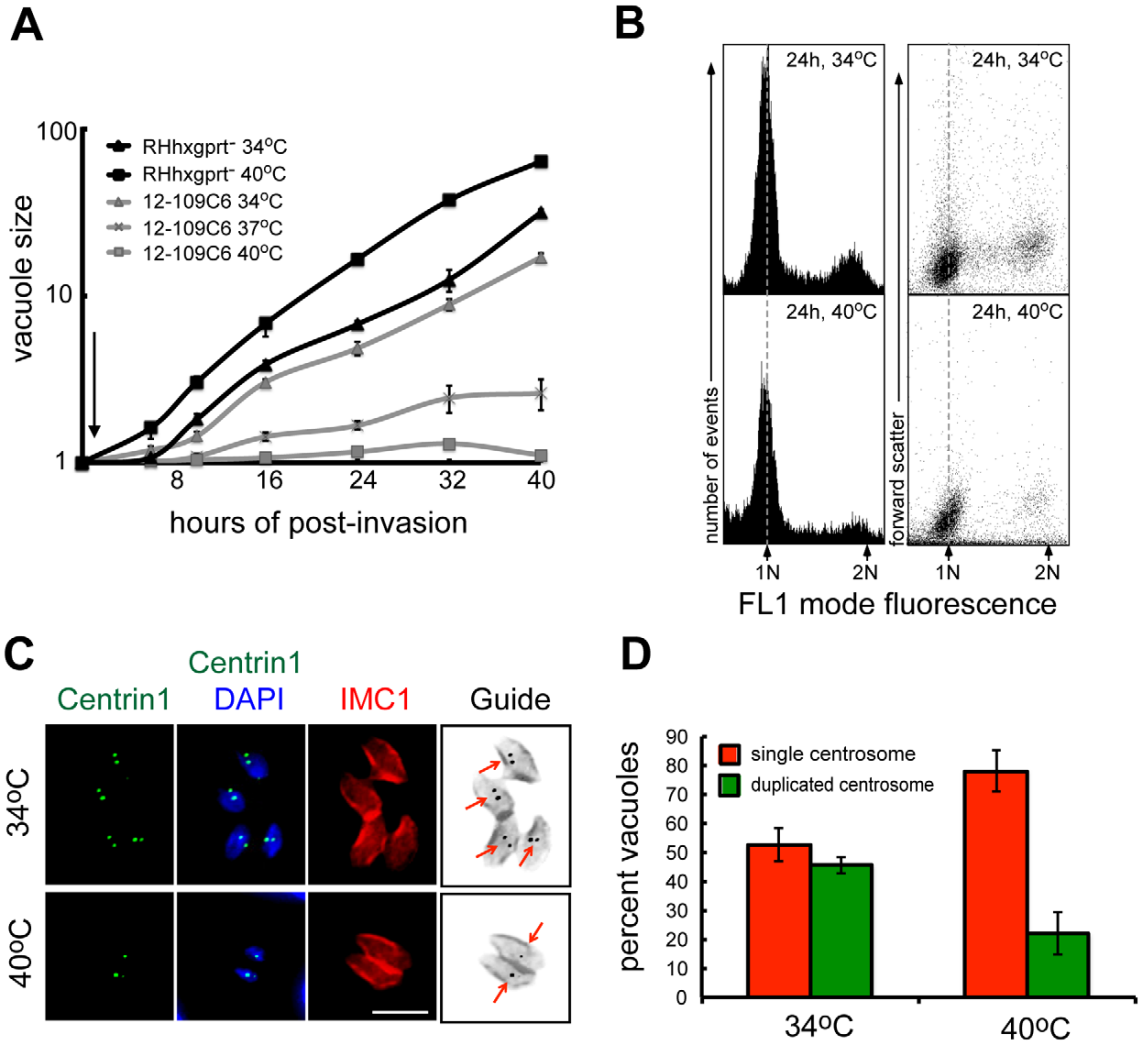
Chemical mutant 12-109C6 conditionally arrests in the G1 phase of the tachyzoite cell cycle. (A) The growth of parental RHΔ*hxgprt* (RHhxgprt-) and mutant 12-109C6 parasites was monitored over a 40 h period (inoculum seed cultures were partially synchronized, see Material and Methods). Following a brief invasion period at 34°C (30 min indicated by arrow) the cultures were maintained at 34°C or shifted to 37°C or 40°C. Average parasite number per vacuole (vacuole size) was determined from 100 randomly selected vacuoles in three independent cultures per strain and temperature condition by direct monitoring live population growth under light microscopy. (B) Mutant parasites grown at 34°C or 40°C for 24 h were stained with SYTOX Green dye and DNA content measured using a FACS calibur (BD Biosciences). The cytometer was set to mode fluorescence and calibrated to asynchronous RHΔ*hxgprt* parental parasites; dashed line references the 1N DNA peak in the histograms. Forward scatter in comparison to DNA content is shown to indicate the distributions of parasite particle size in these populations. DNA fluorescence was measured in FL-1 linear scale (x-axis) and 10,000 events were collected for each histogram. (C and D) Mutant parasites were grown for 24 h at 34°C (upper image panel) or 40°C (lower image panel) and co-stained with anti-human centrin1 (green, centrosomes), anti-IMC1 antibody (red, indicates parasite size and internal daughters), and DAPI (blue, genomic DNA). Labeled marker guide panel is an inverse image of the merged red (IMC1) and green (centrin1) images with arrows indicating duplicated or single centrosomes. Magnification bar is 5 µm. A graph of centrosome counts in mutant 12-109C6 populations grown at 34°C and 40°C for 16 h at the two temperatures were determined in 100 randomly selected vacuoles; single (red bar) or duplicated centrosomes (green bar). The average count was obtained from three independent experiments.

The cell cycle of apicomplexan protozoa has elements in common with other eukaryotes as well as features such as internal daughter budding that are unique to this parasite family. The molecular basis of cell division in these important protists remains understudied compared to other biochemical processes. To expand research efforts in this area, we recently generated a large collection of temperature-sensitive (ts) mutants in order to identify essential mechanisms in *Toxoplasma* replication [8]. An isolate from this collection (mutant 12-109C6) rapidly stopped cell division when shifted to the non-permissive temperature (40°C) indicating a key growth factor was mutated in this parasite. We compared the growth of mutant 12-109C6 to the parental strain (RHΔ*hxgprt*), and while the rate of division in parental parasites increased with temperature, the mutant clone immediately arrested at temperatures above 37°C (Figure 1A). Many mutant parasites at 40°C were unable to complete a single division in the host cell indicating the defect was expressed quickly following temperature shift.

To understand whether clone 12-109C6 was a cell cycle mutant, we examined changes in genomic DNA distributions with respect to incubation temperature. These results revealed the genomic content of the mutant clone grown at 40°C had a higher proportion of haploid (1N) parasites (Figure 1B) consistent with arrest in the G1 phase. Similar to other eukaryotes, *Toxoplasma* tachyzoites duplicate their centrosome at the G1/S boundary [12] providing an internal subcellular marker to further validate the G1 phase arrest (Figure 1C). As expected, mutant parasites incubated at 34°C (Figure 1C, upper panel) duplicated their centrosomes consistent with the known S/M distribution of asynchronous populations (Figure 1D, 48% duplicated) [15]. By contrast, mutant populations exposed to 40°C (lower IFA panel) contained mostly single centrosomes (Figure 1D, 78% singles) and the absence of cytokinesis (*i.e.* internal daughters) in these arrested parasites (Figure 1C) further supports an arrest in the G1 phase. Altogether, these results confirm clone 12-109C6 is a conditional growth mutant carrying a defect in a mechanism needed for G1 to S phase progression.

### The G1 cell cycle arrest of mutant 12-109C6 parasites is linked to a mutation in a unique RNA-recognition-motif protein (TgRRM1)

**Figure 2:**
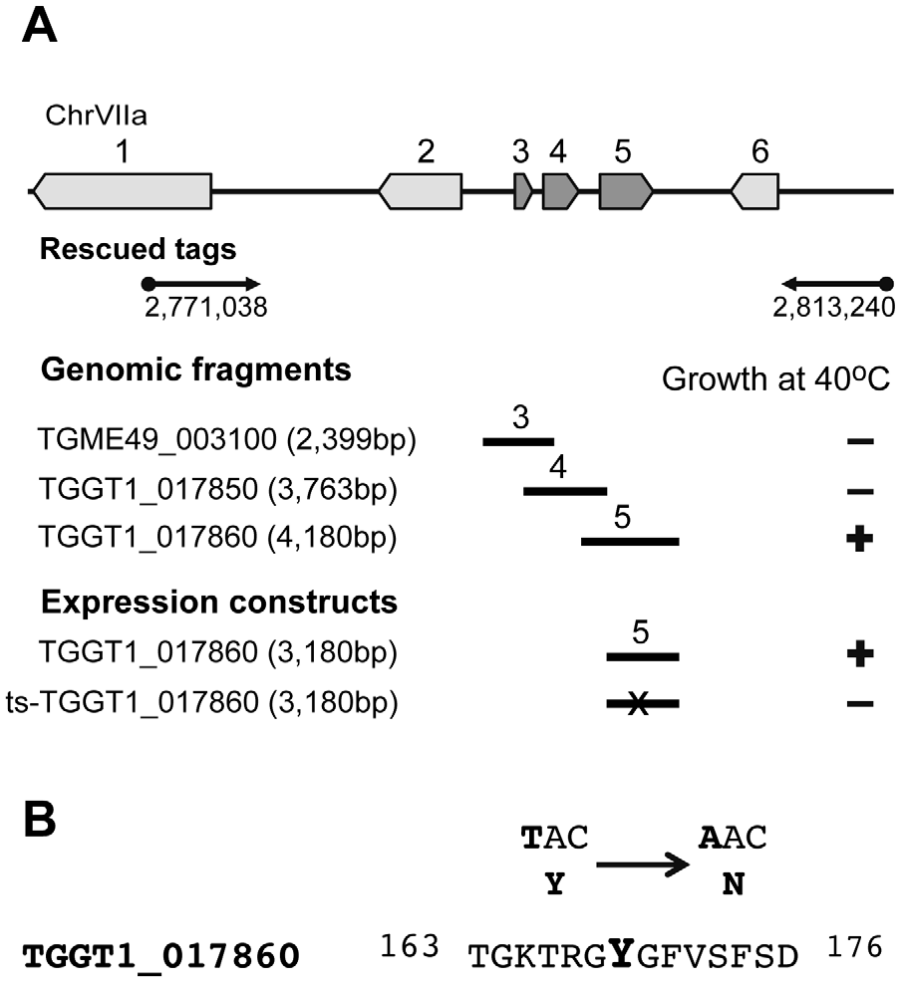
Mutation in a RRM domain-containing protein is responsible for conditional arrest of mutant 12-109C6. (A) Schematic of the chromosome VIIa locus recovered in cosmid insert tags from complemented mutant 12-109C6 parasites (black arrows encompass position 2,771,038 bp to 2,813,240 bp). The locus identified on chromosome VIIa contained six predicted genes (ToxoDB.org). To resolve the locus further mutant 12-109C6 was complemented with genomic fragments containing one of three genes: TGME49_003100 (gene #3, hypothetical protein), TGGT1_017850 (gene #4, hypothetical protein) and TGGT1_017860 (gene #5, RRM domain containing protein). Genomic fragments included the predicted gene promoter, coding region and 3′UTR sequences for each gene tested (see Material and Methods). Note, only gene #5 genomic fragments rescued mutant 12-109C6 parasites from high temperature growth restriction. To confirm the rescue with gene # 5, we prepared ectopic expression constructs based on PCR amplification of gene #5 from the parental or mutant 12-109C6 parasite genomic DNA (Expression constructs = endogenous promoter plus all coding exons fused C-terminally to a triple myc epitope tag). As expected, only gene #5 from the parental strain was able to complement mutant 12-109C6. (B) Sequencing of TGGT1_017860 cDNA from mutant 12-109C6 and parental RHΔ*hxgprt* parasites identified a single transversion mutation (T/A) in the coding sequence of the ts-TgRRM1 allele resulting in a change of tyrosine (Y) 169 to asparagine (N) (see also Figure S1A). Mutated nucleotide and amino acid residue are shown in bold.

We have utilized a forward genetic approach to link the G1 defect in 12-109C6 parasites to the responsible chromosome mutation [8]. Mutant parasites were complemented with *Toxoplasma* cosmid libraries (RH strain genomic DNA) under pyrimethamine selection at 40°C followed by identification of the integrated cosmid insert via marker rescue techniques [8]. Recovered cosmid insert fragments were sequenced and mapped to chromosome VIIa between 2,771,038 bp and 2,813,240 bp (Figure 2A); this chromosome region contains six predicted genes (genes #1-6: TGGT1_017830/dynein 1, beta heavy chain, TGGT1_017840/citrate synthetase, TGME49_003100/hypothetical protein, TGGT1_017850/hypothetical protein, TGGT1_017860/RRM domain-containing protein and TGGT1_017870/conserved hypothetical protein). The locus was resolved to a single gene by a new round of complementation with genomic fragments spanning genes #3, #4, or #5 (Figure 2A). High temperature rescue of mutant 12-109C6 was only observed in parasites transfected with fragments containing gene #5. The protein encoded by gene #5 (TGGT1_017860) is one of 86 genes in the *Toxoplasma* genome predicted to encode proteins with one or more RRM domains (see Table S1 for a full list of RRM containing genes in *Toxoplasma*). The current annotation for gene #5 predicts a 302 amino acid (aa) polypeptide with a single RRM domain flanked by N- and C-polypeptide tails (confirmed by cDNA sequencing, not shown). As the first functionally described RRM protein in *Toxoplasma*, we have designated this gene and protein as TgRRM1. Sequencing of the mutant allele of TgRRM1 (TGGT1_017860) revealed a single thymidine to adenine transversion at nucleotide position 505 of the coding sequence that led to exchange of tyrosine 169 for an asparagine residue (Figure 2B). The mutation lies in the RRM domain affecting one of the conserved aromatic residues in the RNP1 subdomain predicted to involve RNA binding (Figure S1A). A plasmid construct expressing temperature-sensitive allele of gene #5 (see Dataset S1 and Material and Methods for all construct designs) failed to rescue mutant 12-109C6 at 40°C confirming the Y/N non-synonymous mutation in this protein is responsible for temperature sensitivity (Expression constructs, Figure 2A).

**Figure 3:**
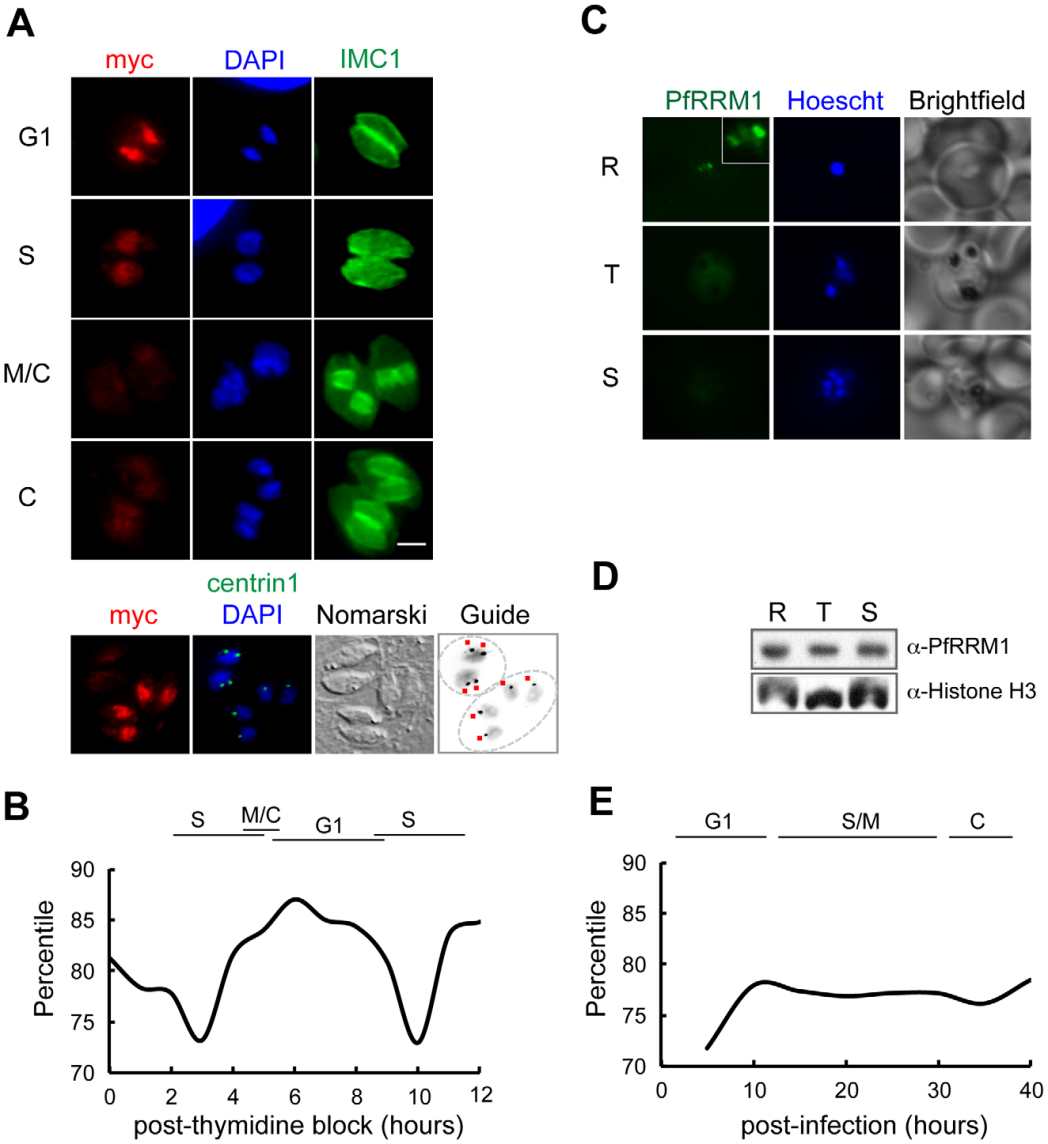
Expression of TgRRM1 is cell cycle–dependent. (A) A genetically rescued mutant 12-109C6 clone expressing wt-TgRRM1^myc^ under control of the native TgRRM1 promoter was evaluated for cell cycle expression. Parasites were grown for 24 h at 34°C and then processed for IFA by co-staining with anti-myc (green = TgRRM1^myc^ protein), anti-IMC1 (red) and DAPI (blue) as in Figure 1. Four image panels (G1 to C phases) show the basic cell cycle profile of wt-TgRRM1^myc^ expression. Magnification bar (2 µm) is shown. Note that intravacuolar parasites were tightly synchronized allowing the cell cycle position of each vacuole (defined on the left) to be assigned based on known characteristics [6], [7], [49]. Peak expression of wt-TgRRM1^myc^ was observed in the G1 panel, while the protein was nearly undetectable in parasites undergoing cytokinesis (M/C and C panels) demonstrating this factor is tightly cell cycle regulated. A fifth image panel (bottom) is included that pinpoints wt-TgRRM1^myc^ expression with respect to the G1 and S transition; co-staining in the panel is red = anti-myc, green = anti-centrin1, and blue = DAPI. The marker guide panel included here is an inverse image of the merged blue (DAPI) and green (centrin1) images to highlight the centrosome content marked by adjacent red dots. Note, parasites in these two separate vacuoles have single nuclei with no internal daughters, which places their cell cycle position on either side of the G1/S boundary based on single versus double centrosomes (G1 versus S phase, respectively). Strong wt-TgRRM1^myc^ expression was detected in the vacuole where parasites possessed a single centrosome (vacuole of 4 in G1), whereas wt-TgRRM1^myc^ was downregulated in S phase parasites associated with recently duplicated centrosomes (vacuole of 2). (B) Cyclical profile of TgRRM1 mRNA spanning nearly two tachyzoite division cycles also shows G1 phase peak expression. The graph is based on expression values obtained from our *Toxoplasma* cell cycle transcriptome microarray dataset [13]. (C) Immunostaining of *Plasmodium falciparum* merozoites shows distinct cell cycle distribution of PfRRM1 in the nucleus of the ring stage parasites. While the protein is detected in discrete nuclear bodies in ring stages (see Inset), it appears diffused in the nuclei and cytoplasm of trophozoites and schizont stages and barely detectable by IFA. (R- ring; T- trophozoite; S- schizont; Hoechst- nucleic acid stain). (D) A time course immunoblot analysis of *P. falciparum* ring (R) (8–16 hours post-invasion), trophozoites (T) (24–32 hours post-invasion), and schizont (S) (36–44 hours post-invasion) stages shows constitutive overall expression of PfRRM1 throughout the intraerythrocytic cycle. Anti-Histone H3 antibody was used as a loading control. (E) The graph represents the percentile value of PF13_0318 mRNA measured in the synchronized population of *P. falciparum* 3D7 [50].

The rapid and lethal growth arrest of mutant 12-109C6 suggests TgRRM1 has a vital, if unknown role in cell division. To build clues to function, we first explored how TgRRM1 is expressed in the parasite cell cycle by introducing an epitope tagged version (wt-TgRRM1^myc^) controlled by the native TgRRM1 promoter (primers, constructs and strains, Dataset S1 and Material and Methids). The wt-TgRRM1^myc^ protein rescued mutant 12-106C6 at 40°C (reported at the bottom of Figure 2A) where we observed maximum expression of the factor concentrated in the nucleus of G1 parasites before protein levels dropped below detection in parasites that were undergoing mitosis and early cytokinesis (Figure 3A). The cell cycle profile of wt-TgRRM1^myc^ followed closely the cyclical timing of the mRNA encoding the native protein that also peaked in G1 (Figure 3B) [13]. Importantly, wt-TgRRM1^myc^ downregulation coincided with centrosome duplication, which is thought to mark commitment to DNA replication and entry into S phase [16]. The tight “on-off” cell cycle switching of wt-TgRRM1^myc^ was clearly evident in representative vacuoles (Figure 3A, bottom 4 image panel) containing parasites with single versus duplicated centrosomes. In this single microscopic field, wt-TgRRM1^myc^ was expressed only in the cells on the G1 side of the G1/S transition. It is important to note that the cell cycle profile of TgRRM1 likely results from transcriptional and post-transcriptional mechanisms as the encoded mRNA levels never fall below the 70^th^ percentile in the cell cycle transcriptome data (Figure 3B), while protein levels are clearly more dynamic based on IFAs (Figure 3A). These observations are similar to other cell cycle proteins we have studied in tachzyoites [13].

To understand whether the cell cycle timing of this factor was conserved in other Apicomplexa, we raised antiserum against the *Plasmodium falciparum* ortholog (PF13_0318, designated PfRRM1). The specificity of this antiserum and cross-reactivity to TgRRM1 was verified by Western analysis (Figure S2). Western and mRNA analysis of synchronized *P. falciparum* merozoites demonstrated that PfRRM1 is constitutively expressed (Figure 3D and 3E), which is different from the periodic profile of TgRRM1. The cell cycle of the *P. falciparum* merozoite also has an early G1 phase termed the ring form, and it was in this well recognized stage we detected PfRRM1 concentrated in discrete intranuclear bodies (Figure 3C). PfRRM1 was diffusely distributed in the nucleus of the trophozoite or schizont stages that are the equivalent to S and M/C phases, respectively (Figure 3C). It is possible that in *P. falciparum* nuclear redistribution is the major cell cycle feature of this related factor.

**Figure 4:**
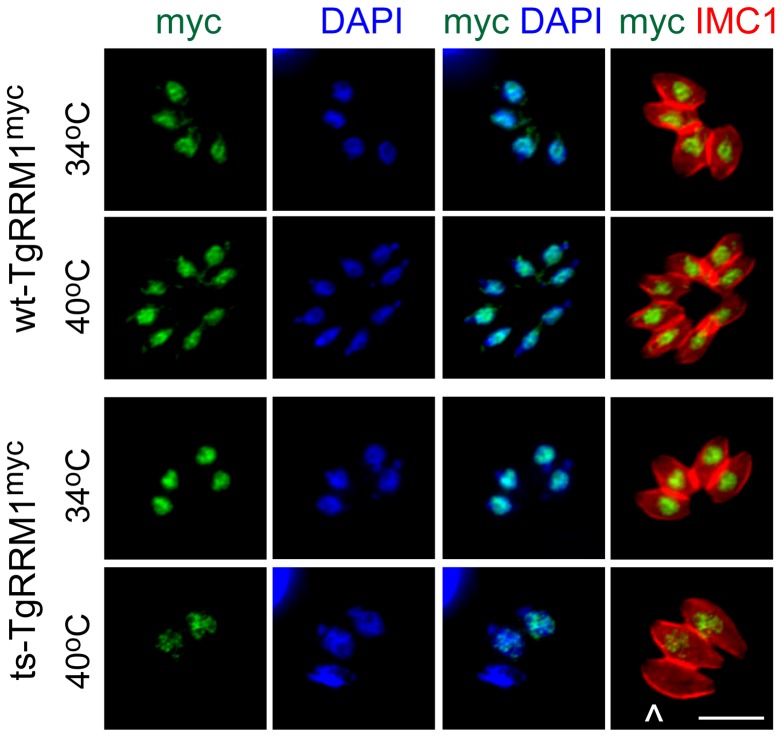
Loss of TgRRM1 expression underlies the temperature lethal phenotype of mutant 12-109C6. Expression of epitope tagged wt-TgRRM1^myc^ or ts-TgRRM1^myc^ proteins (C-terminal fusion of myc_3X_) under the native promoter in mutant 12-109C6. Transgenic clones were grown at 34°C or 40°C for 24 h, harvested for IFA, and then co-stained using anti-myc = green, anti-IMC1 = red, and DAPI = blue as in Figure 3. In order to compare protein expression, fluorescent micrographs were collected under identical exposure and processing conditions. Magnification bar (5 µm) is shown. The arrow in the bottom image series (myc/IMC merge) indicates a parasite lacking ts-TgRRM1^myc^ expression that had failed to divide at 40°C.

Finally, we explored whether the molecular basis for the mutant 12-109C6 cell cycle defects was caused by changes in either the expression and/or alterations in the cellular localization of the temperature sensitive TgRRM1 protein (ts-TgRRM1^myc^). We confirmed ts-TgRRM1^myc^ was unable to rescue mutant 12-109C6 at the high temperature employing a plasmid construction based on native promoter expression as the wt-TgRRM1^myc^ construct used above (see Expression constructs, Figure 2A). Immunostaining with anti-myc antibody demonstrated both TgRRM1^myc^ isoforms (wt versus ts) were concentrated in the nucleus (see merged anti-myc and DAPI images, Figure 4), although the level of the ts-TgRRM1^myc^ protein was significantly reduced at the restricted temperature indicating the Y to N change may primarily affect protein stability. In the single vacuole of three parasites in the bottom panel (24 h at 40°C) there was a loss of intravacuolar synchrony that correlated with differential ts-TgRRM1^myc^ expression; the parasite negative for ts-TgRRM1^myc^ had not divided, whereas the other two other parasites still positive for ts-TgRRM1^myc^ had progressed into the second cell cycle following invasion. Thus, the timing of TgRRM1 loss at high temperature likely determines whether a parasite arrests within the G1 period of the present or the next division cycle. Due to the conserved protein sequence, the antiserum raised against recombinant PfRRM1 also binds TgRRM1, and this reagent was used to confirm the instability of the encoded ts-TgRRM1 protein (in mutant 12-109C6) at the restrictive temperature (Figure S2). Western analysis showed the original 12-109C6 mutant parasites lost ts-TgRRM1 protein quickly upon shift to 40°C (Figure S2).

### Deletion analysis of TgRRM1 defines minimal structures required for temperature rescue

**Figure 5:**
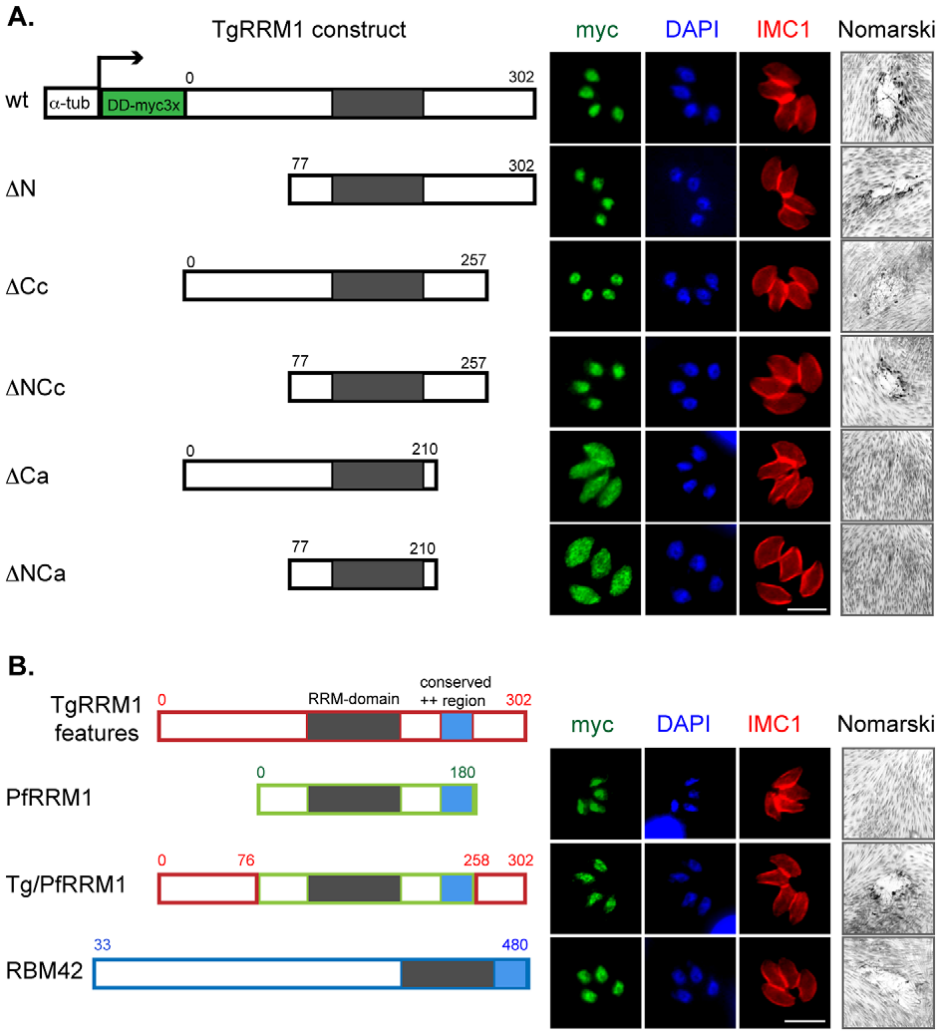
Structure-function characterization of TgRRM1. (A) The designs of wild type and five TgRRM1 deletion constructs are shown. All polypeptides were expressed under control of the α-tubulin promoter and a DDmyc_3X_ tag was fused to the N-terminal end of each protein for visualization (DD = FKBP destabilization domain) [46]. The residue numbers refer to the proportion of TgRRM1 coding included in each construct. Note omitting shield 1 reduced wt-TgRRM1^DDmyc^ protein levels ∼3-fold, however, this minimal change was not sufficient to prevent genetic complementation (not shown). Thus, representative results of parasites cultured in standard culture media plus 100 nM shield 1 are shown only. Immunofluorescent images of stable transgenic clones expressing the corresponding constructs on the left were developed as in Figure 3. The right panels show Nomarski images of the plaques formed by the transgenic parasites grown at 40°C. Magnification bar (5 µm) is shown. Note that genetic complementation failed only in constructs (ΔCa and ΔNCa) where C-terminal residues 210 to 257 were deleted. (B) *P. falciparum* PfRRM1 and human RBM42 are functional orthologs of *Toxoplasma* protein TgRRM1. Schematic description of PfRRM1 and RBM42 expression constructs are indicated along with the TgRRM1 reference. The construction of these expression plasmids followed the designs described for TgRRM1 above and were transfected into mutant 12-109C6 parasites. Numbered residues indicate the start and end of each polypeptide. Red boxes = polypeptide of TgRRM1 origin, green = PfRRM1 regions, and blue = RBM42 regions. Immunofluorescent images were from transgenic clones cultured for 24 h in standard media plus 100 nM shield 1. The co-stains were myc tagged PfRRM1, Tg/PfRRM1 chimera, or RBM42 transgene expression (green = anti-myc), red = anti-IMC1, blue = DAPI. Nomarski images on the right show plaques formed by the clones cultured for 7 days at 40°C (no shield 1). Chimeric Tg/PfRRM1 and human RBM42 constructs were able to rescue high temperature sensitivity of mutant 12-109C6, while PfRRM1 delayed the growth arrest of the mutant (not shown) but this was not sufficient to support long term plaque formation.

The single RRM domain in TgRRM1 may provide interaction with nucleic acids, as this class of proteins is known to bind ssRNA or ssDNA [17], [18]. The RNA binding domain of TgRRM1 can be readily modeled (Figure S1B) into one of the resolved RRM folds [18] suggesting TgRRM1 likely also binds RNA. Yet, the protein features required for cellular replication are the key functional question, which we explored by determining the minimal TgRRM1 structures able to complement mutant 12-109C6 (see Figure 5 and Dataset S1 for construct designs). It is assumed the RRM domain is critical to function based on the ts-mutation, therefore our deletion study focused on the extended N- and C-terminal tails. Interestingly, TgRRM1 deletions (TgRRM1^DDmyc^ series) that truncated the first 76 amino acids (ΔN, 77–302 aa) or removed 45 amino acids from the C-terminal end (ΔCc, 1–257 aa) were fully capable of rescuing the mutant 12-109C6 at high temperature (Figure 5A). Likewise, we found combining these deletions in a single construct design (ΔNCc, 77–257aa) were also functional indicating the N- and most distal C-terminal residues were dispensable for genetic rescue of mutant 12-109C6. The deletion of an additional 47 residues (total deletion of 92 residues) in the C-terminal tail with or without a N-terminal deletion (ΔCa or ΔNCa) failed to complement mutant 12-109C6, and also changed the protein subcellular distribution. The ΔCa- and ΔNCa-TgRRM1^DDmyc^ proteins were not excluded from the nucleus but also did not concentrate there suggesting residues 210-257 of the TgRRM1 C-terminal tail either carry a signal for nuclear retention or alternatively the loss of function indirectly causes the observed change in cellular distribution.

### The critical cell cycle functions of TgRRM1 are evolutionarily conserved

TgRRM1 orthologs can be found by mining genomic sequence of other eukaryotic species, including all Apicomplexa for which there is sequence available, but also in multicellular plants and animals (see Figure S3 for protein alignment). The overall similarity in this protein family is moderate with the RRM domain showing the highest conservation (80–90% similarity), while N- and C- terminal extensions when present are typically unique even in orthologs from related species. Secondary structure analysis of divergent TgRRM1 orthologs illustrates protein similarity beyond the primary sequence (Figure S1C) including the positively charged region C-terminal to the RRM domain we found was critical for function and nuclear retention (Figure 5A). The mutant 12-109C6 offers a unique opportunity to explore whether these minimal structures conserved in TgRRM1 orthologs are sufficient for function. We tested PfRRM1 from the related apicomplexan *P. falciparum* and RBM42 from human cells (see construct designs Figure 5B). The *P. falciparum* PfRRM1 differs from TgRRM1 by having shorter N- and C-terminal tails flanking the central RRM domain. PfRRM1^DDmyc^ was unable to fully rescue mutant 12-109C6, although expression levels of PfRRM1^DDmyc^ and nuclear retention (Figure 5B) were comparable to wt-TgRRM1^DDmyc^ (Figure 5A). Interestingly, the mutant isolates expressing PfRRM1^DDmyc^ did not immediately growth arrest at 40°C like the original ts-mutant strain (Figure 1) suggesting PfRRM1^DDmyc^ was partially complementing the defect (not shown). We then added the *Toxoplasma* N- (0–76 residues) and C-terminal (258–302 residues) tails to the core PfRRM1 domain (see Figure S4 for chimera designs) and achieved full genetic rescue of mutant 12-109C6 with the chimeric protein Tg/PfRRM1^DDmyc^ (Figure 5B). These results are consistent with the conservation of the *Toxoplasma* and *Plasmodium* RRM domains (93% similarity/70% identity, Figure S3), although they also demonstrate nuclear retention alone is not sufficient to achieve functional complementation. Human RBM42 shares 64% identity and 89% similarity to TgRRM1 in the RRM domain. RBM42 has an extended N-terminus that is structurally similar to the N-terminal extension of TgRRM1 in the residues immediately upstream of the RRM domain (Figure S1C). The slightly shorter C-terminal tail of RBM42 nonetheless preserves the charged nuclear retention domain. Remarkably, RBM42^DDmyc^ protein (residues 33–480 aa) was able to fully rescue the 12-109C6 mutant at high temperature with a restoration of a wild type growth rate equivalent to complementation with wt-TgRRM1^DDmyc^ (Figure 5B). Rescue of ts-TgRRM1 mutants was specific for RBM42 as genetic complementation of mutant 12-109C6 failed using the non-homologous human RRM proteins, CCR4-NOT transcription complex subunit 4 (NP_037448.2) and eukaryotic translation initiation factor 3 (NP_003742.2), despite the proper localization of the CCR4-NOT factor to the parasite nucleus (data not shown).

### TgRRM1 is a novel regulator of mRNA splicing

**Figure 6:**
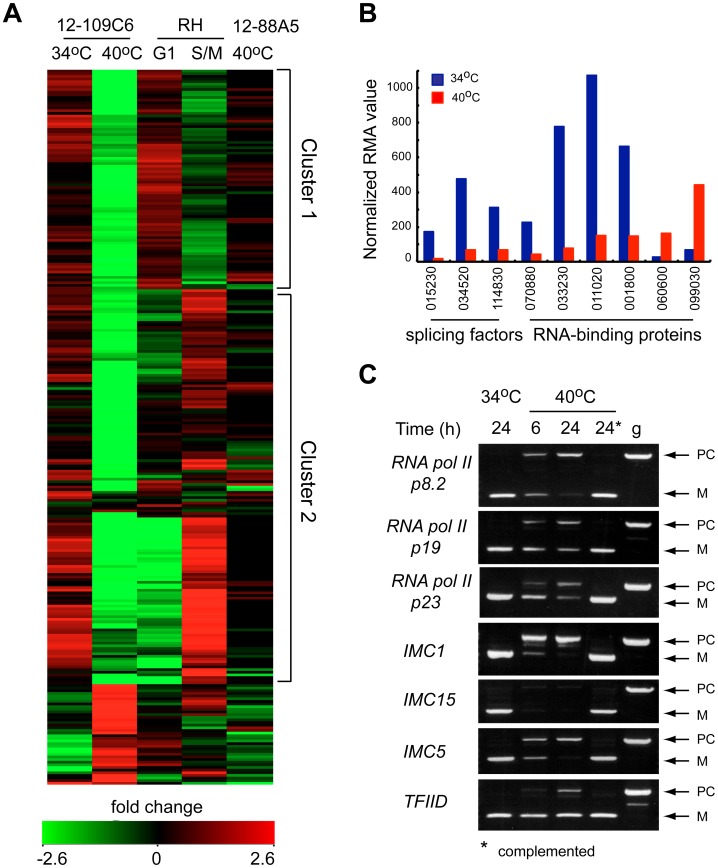
Loss of TgRRM1 in mutant 12-109C6 leads to significant downregulation of mRNAs as the result of mis-splicing. (A) Microarray analysis identified cell cycle regulated mRNAs (261 total) that were altered by temperature in mutant 12-109C6 (24 h incubation at 34°C = lane 1 versus 40°C = lane 2). The heat map displays the microarray results for the 261 mRNAs in comparison to their expression in the established tachyzoite cell cycle transcriptome [13] and in an unrelated G1 mutant (lane 5). Reference data from 0–12 h thymidine-synchronized populations; 6 h time point shows G1 peak expression for mRNAs in cluster 1 (lane 3 in the heat map), while the 3 h time point from this series corresponds to S/M peak expression (lane 4 and cluster 2). Note, inclusion of microarray results (lane 5) from a second G1 mutant also growth restricted at 40°C (ts-mutant 12-88A5) [13] confirms that mRNA changes in mutant 12-109C6 are not the result of non-specific temperature restriction or a general pattern of G1-specific arrest. (B) The relative mRNA abundance (normalized RMA values) for splicing factors and RNA binding proteins affected by temperature restriction in mutant 12-109C6. Gene ID numbers are shown minus the common TGME49_ label (see ToxoDB.org). (C) PCR analysis of mRNA splicing for seven selected genes. Total RNA purified from the mutant 12-109C6 cultured at 34°C (24 h), at 40°C (6 and 24 h) and mutant 12-109C6 complemented with wt-TgRRM1^myc^ cultured at 40°C (24 h, indicated with asterisk) were reverse transcribed and PCR amplified using primers that span an intron. PCR of genomic DNA was included as a reference to distinguish between properly spliced (M) and pre-spliced (PC) forms of each gene. The following genes were analyzed: RNA polymerase II p8.2 subunit (TGGT1_028500), RNA polymerase II p19 subunit (TGGT1_110170), RNA polymerase II p23 subunit (TGGT1_050280), IMC1 (TGGT1_116030), IMC15 (TGGT1_000660), IMC5 (TGGT1_079150), and transcription factor IID (TGGT1_011510).

The rapid cell cycle arrest of mutant 12-109C6, and the potential of TgRRM1 to bind RNA, led to us to look for clues to TgRRM1 function in whole-cell gene expression. Total RNA was isolated in duplicate from mutant 12-109C6 parasites grown at permissive and non-permissive temperatures, converted to cRNA and used to hybridize a custom Affymetrix GeneChip with multiple probes for ∼8,000 *Toxoplasma* genes (http://ancillary.toxodb.org/docs/array-tutorial.html). A total of 473 mRNAs was statistically altered (fold change ≥4 up or down) when mutant 12-109C6 was shifted to the higher temperature. Most transcriptome changes involved decreases in mRNA levels occurring by 6 h post-temperature shift and affected many different pathways of cell metabolism (see Dataset S2 for full gene list). Our recent analysis of the *Toxoplasma* cell cycle transcriptome identified two major waves of transcription with peak mRNA levels associated with G1 or S/M subtranscriptomes [13]. In comparison to this cell cycle transcriptome, we found 261 mRNA profiles altered in the 12-109C6 mutant that were also periodic mRNAs in the tachyzoite division cycle. A heat map of the 261 mRNAs reveals both G1 and S/M transcripts are included (Figure 6A; G1 mRNAs peak in cluster 1, S/M mRNAs peak in cluster 2), and in nearly every instance, these mRNAs from either half of the cell cycle were strongly downregulated in temperature restricted 12-109C6 parasites (Figure 6A compare 34°C versus 40°C). A reduction in the levels of mRNAs that peak in the tachyzoite S/M periods was expected given the G1 arrest of mutant 12-109C6, however, reductions of G1 peak transcripts were a surprise. Importantly, mRNA levels were not extensively downregulated in asynchronously dividing 12-109C6 parasites (grown at 34°C) (Figure 6A, lane 1) or in an unrelated mutant (ts-mutant 12-88A5) previously shown to rapidly arrest in the G1 phase at 40°C (Figure 6A, lane 5) [8], [13].

In the set of altered mRNAs in mutant 12-109C6, a few transcripts encoded proteins with possible functions related to TgRRM1 (Figure 6B). Among the abundant family of the novel RNA binding proteins in *Toxoplasma* (86 total, Table S1), four mRNAs were downregulated, while two were upregulated (Figure 6B, TGME49_ numbers of these genes are indicated). Two proteins associated with the U2 spliceosome and the splicing factor 3b subunit 10 were also significantly downregulated (Figure 6B). To examine whether splicing was affected in mutant 12-109C6 parasites at high temperature, we surveyed a selection of genes including key RNA polymerase II subunits using primers spanning an intron (Figure 6C). Semi-quantitative PCR analysis showed the accumulation of pre-mRNA for all genes, which was readily detected as early as 6 h following the shift of 12-109C6 parasite cultures to 40°C (Figure 6C, lane 6 h). Pre-mRNA was not amplified from total RNA obtained from 12-109C6 parasites grown at 34°C or from 12-109C6 parasites complemented with wt-TgRRM1^myc^ grown at 40°C (Figure 6C, lane 24*). The stability of unspliced mRNA was variable with pre-mRNA levels equal to the matched spliced mRNA for some genes, whereas pre-mRNA levels were significantly lower than the mature mRNA in others.

**Figure 7:**
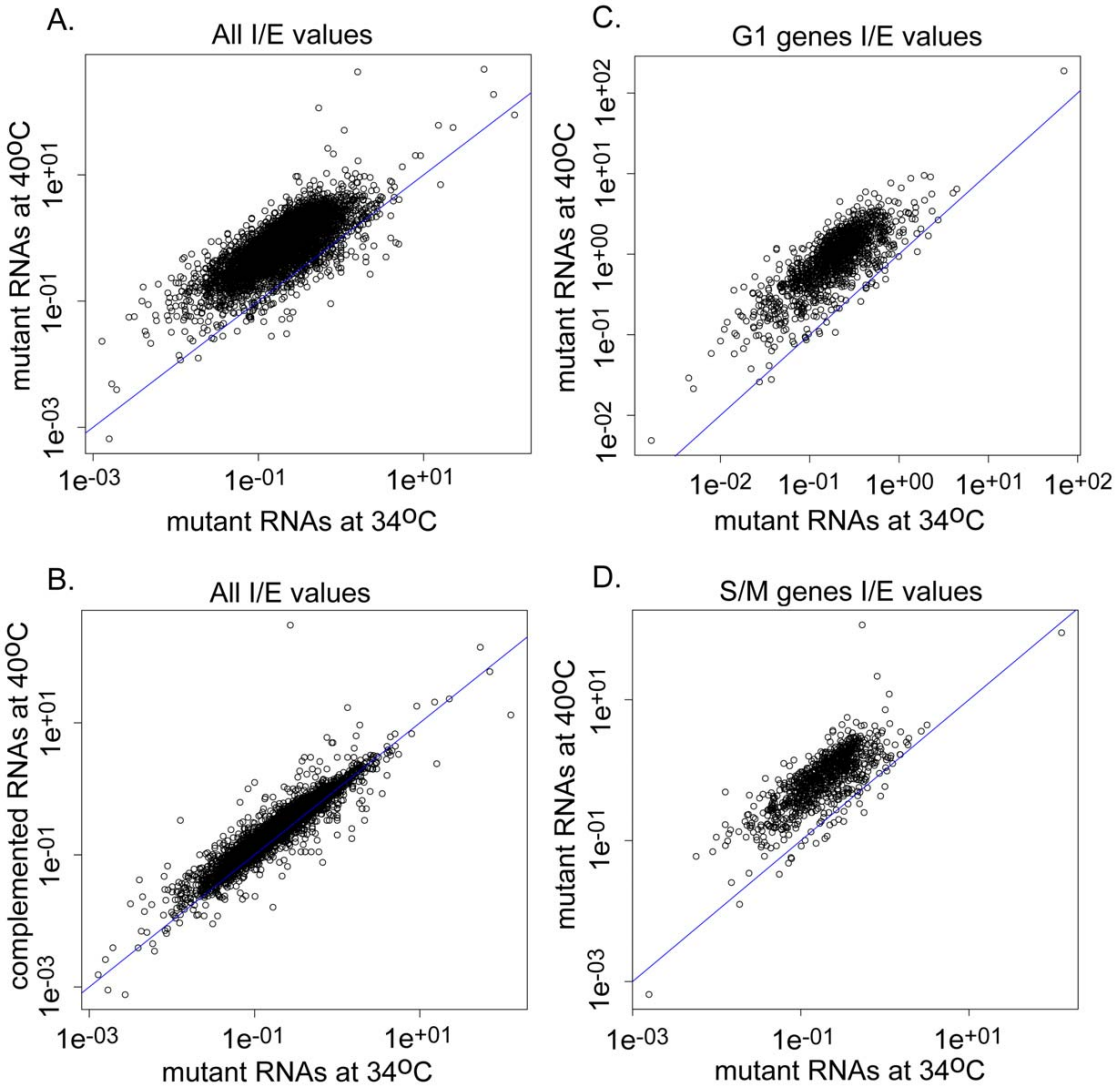
Splicing defects in TgRRM1 null parasites occur across the parasite transcriptome. Splicing of mRNAs is globally disrupted in 12-109C6 parasites grown at 40°C (null for TgRRM1, see Figure 4). (A) Intron∶exon (I/E) values for genes expressed in 12-109C6 mutant parasites grown at the permissive temperature (34°C, x-axis) compared to the non-permissive temperature (40°C, y-axis). (B) I/E ratios for genes from the permissive condition (x-axis) compared to those of a genetically complemented strain at the non-permissive temperature (y-axis). (C) As in (A), but selecting only those genes with peak expression in the G1 phase of the cell cycle [13]. (D) As in (A), but selecting only those genes with peak expression in the S and M phases of the cell cycle. All axis values are plotted in log scale. Note that the axis scale reflects the inherent range of intron/exon content and length for the 5,833 genes in this dataset.

The accumulation of unspliced mRNA would contribute to hybridization signals on the microarrays suggesting we may have underestimated the influence of TgRRM1 on global gene expression. We have addressed this question by deep sequencing RNA samples from 12-109C6 parasites and the complemented strain (Figure 7, Table S2 and Dataset S3). We calculated the total number of RNA reads aligning to either exons or introns (based on ToxoDB 6.1 predictions) and determined the ratio of intronic to exonic hits (I/E) for each spliced gene under conditions of mutant parasites grown at 34°C versus 40°C, and determined whether genetic rescue restored I/E ratios at the higher temperature (Figure 7 and Table S2). A spreadsheet listing the I/E values for the 5,833 intron-containing genes under the conditions examined along with the overall abundance levels for all RNAs detected (including single exon genes) is included in Dataset S3. As expected, the overall I/E ratios were dramatically increased when mutant 12-109C6 parasites were shifted to 40°C (Figure 7A). This effect was genome-wide (total of 5,204 mRNAs affected, Dataset S3) and is consistent with a global defect in mRNA intron splicing. Few genes showed an increase in splicing (184 total) and these were largely very low expressed and enriched for genes expressed in other developmental stages. A few genes with decreased I/E ratios had wrong gene models (not shown). Thus, TgRRM1 appears to be a factor required for general mRNA splicing, and consistent with this view, the genetic rescue of the splicing defect at high temperature was nearly complete (Figure 7B, >98% of I/E ratios in the 40°C complemented sample were fully or partially restored to the values observed in the 34°C RNA samples). There was a statistically nonsignificant (p>0.05) increase in the complemented strain I/E ratios grown at 40°C compared to the mutant grown at 34°C (Table S2A).

The I/E ratios of genes shown to be regulated during the course of the cell cycle were also examined. Genes were categorized as being either up-regulated during either chromosome synthesis and mitosis (S/M) phases (1,217 genes) or during growth in the G1 phase (1,635 genes) [13]. In general, I/E values of G_1_ and S/M phase genes behave in much the same way as the general population, increasing significantly when grown at 40°C, but recovered by complementation with the TgRRM1 wt-allele (Figure 7C and 7D, Table S2B). Due to the essential nature of this mechanism to mRNA splicing, we were not surprised to find steady state levels for all genes, including those without predicted introns, are impacted by this defect (Figure S5 and Dataset S3). There is a much larger amount of variance in the mutant parasites grown at 40°C than the parasites grown at 34°C (Figure S5A). In overall agreement with the microarray results (Figure 6), mRNA expression was severely reduced following the shift of mutant parasites to the non-permissive temperature. This was true for cell-cycle dependent genes as well single exon genes (Figure S5B). The reduction in mRNA was reversed by complementation of the mutant with the wt-TgRRM1 allele (Figure S5A and S5C).

### TgRRM1 associates with the U4/U6.U5 tri-SNP complex required for assembly of the spliceosome

**Figure 8:**
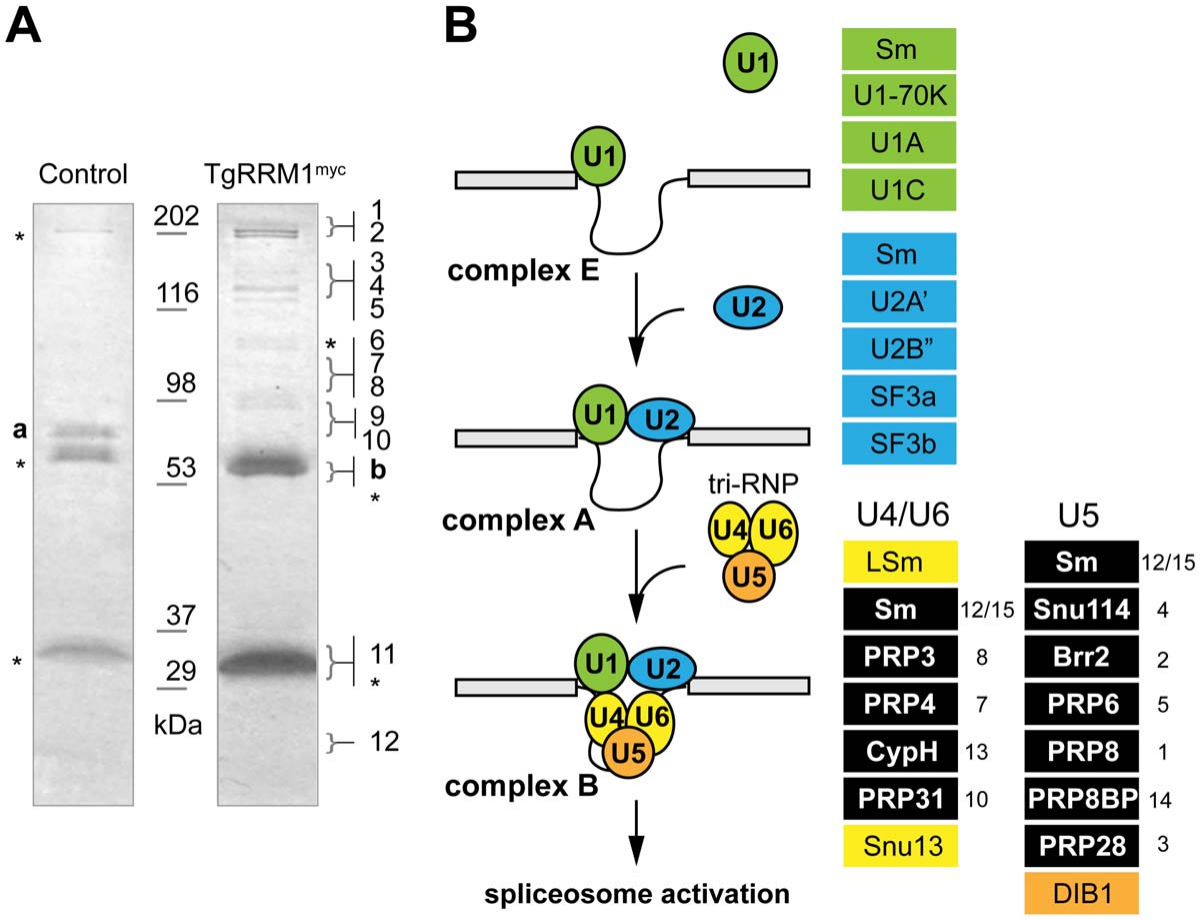
TgRRM1 is associated with U4/U6.U5 subcomplex of the spliceosome. (A) TgRRM1 was immunoprecipitated with anti-myc antibody from the whole cell lysates (shown here) or nuclear extracts (Dataset S4) of the transgenic clone 12-109C6 expressing wt-TgRRM1^myc^. The original mutant strain 12-109C6 was used as a negative control. Eluates from experimental (wt-TgRRM1^myc^) and control immunoprecipitations were resolved on SDS-PAGE and stained with Commassie Blue. Digestion of the gel slices and mass-spectrometry analysis of protein peptides is described in Material and Methods. Proteins identified in the series of gel slices marked by braces (}) are numbered here and in the splicing diagram below. These numbers also correspond to the protein list in Table 1. Stars indicate host cell protein contamination. TGME49_119920 (“a”) was a single protein identified in the negative control pull-down. The highly abundant ROP1 protein (TGME49_109590, “b”) was also found in these experiments and is a common contaminant in *Toxoplasma* co-IP/proteomic analyses. Molecular mass standards are indicated between the gel lanes. (B) Schematic diagrams spliceosome assembly comprising the stepwise incorporation of U1 (green), U2 (blue), U4/U6 (yellow) and U5 (orange) snRNP complexes to intronic sequences of pre-mRNA. Core proteins in each snRNP complex are shown in the color boxes on the right of the scheme. Splicing factors co-precipitated with TgRRM1 belonged to U4/U6 or U5 snRNPs and are denoted by the black boxes in each column. Numbers on the right of the boxes correspond to the protein number in the gel slices in (A) and to the proteins listed in Table 1. A complete list of identified proteins is included in Dataset S4.

The global mRNA splicing defect caused by TgRRM1 downregulation in mutant 12-109C6 raised the possibility this factor has a direct involvement in spliceosome function. The cellular components that assemble into active splicing machinery include a number of RRM proteins, although there is no report of RBM42 or its orthologs serving as an integral component of the splicing machinery. To explore this possibility, co-immunoprecipitation followed by a comprehensive proteomic analysis was performed on a temperature resistant clone rescued by wt-TgRRM1^myc^ complementation (Figure 2A) and compared to the original 12-109C6 mutant as a negative control. Purified complexes from whole cell lysates (Figure 8A) or nuclear extracts (see Dataset S4) were resolved by electrophoresis and individual gel slices subjected to mass spectrometry analysis. These experiments identified 14 and 21 unique proteins in the whole lysate and nuclear extracts, respectively, including TgRRM1 itself (see Dataset S4). Proteins interacting with TgRRM1 are encoded by mRNAs with a wide range of abundance from low expression (50^th^ percentile) to highly abundant transcripts (90^th^ percentile) (see Table 1 for examples). TgRRM1 containing complexes were highly enriched for spliceosome factors with multiple peptides recovered and up to 12% sequence coverage for some splicing factors; 15 out of 23 co-precipitated proteins are known components of the U4/U6 or U5 small ribonucleoprotein particles (see Dataset S4 for full details). Five core components of U4/U6 snRNP, seven components of U5 snSNP, and three accessory proteins were identified (all indicated in solid black in Figure 8B). Few proteins and no splicing factors were identified in negative controls. The 14 splicing factors identified in these pull-downs including the 10 proteins recovered from both extracts are listed in Table 1. The selective co-precipitation of TgRRM1 with U4/U6 and U5 snRNPs suggests a role for this protein in spliceosome function at the level of complex B formation (Figure 8B).

**Table 1:** Summary of spliceosome factors co-immunoprecipitated with TgRRM1.

Protein hit	IP fraction^a^	Gene ID^b^ TGME49_	Gene product	% mRNA^c^	mRNA peak^d^	RBM42 interaction^e^	Splicing subcomplex associations	References^f^
1	WCL/NE	031970	PRP8	70	n/p	no	U5 snRNP-associated protein	www.genome.jp/kegg/pathway
2	WCL/NE	023390	Brr2	60	G1	no	U5 snRNP-associated helicase	Achsel et al., 1998
3	WCL/NE	098020	PRP28	90	G1	no	U5 snRNP-associated helicase	Mathew et al., 2008
4	WCL/NE	086080	Snu114	60	G1	no	U5 snRNP-associated GTPase	Fabrizio et al., 1997
5	WCL/NE	005220	PRP6	50	n/p	no	Connects U5 and U4/6 snRNPs	Laggerbauer et al., 2005
6	WCL	118140	SART1	60	G1	no	U4/U6.U5 tri-snRNP-associated	Stanek, Neugebauer, 2006
7	WCL/NE	043540	PRP4	50	G1	yes	U4/U6 snRNP-asociated	www.genome.jp/kegg/pathway; http://thebiogrid.org/; Sowa et al., 2009
8	WCL/NE	019790	PRP3	80	G1	no	U4/U6 snRNP-asociated	www.genome.jp/kegg/pathway
9	WCL/NE	094360	Sad1	80	G1	yes	U4/U6.U5 tri-snRNP-associated protein ubiquitin hydrolase	Makarova et al., 2001; Sowa et al., 2009
10	WCL	044100	PRP31	65	N/A	no	U4/U6 snRNP-asociated	www.genome.jp/kegg/pathway
11	WCL/NE	072010	snRNP	70	G1	no	Ortholog of GAR1, predicted interaction with Sad1	http://thebiogrid.org
12	WCL/NE	109740	SmD3	60	G1	no	snRNP core protein	Kambach et al., 1999
13	NE	085760	CypH	60	G1	no	U4/U6 snRNP-asociated	www.genome.jp/kegg/pathway
14	NE	110860	PRP8BP	60	G1	no	U5 snRNP-associated protein	www.genome.jp/kegg/pathway
15	NE	100280	Sm	80	G1	no	snRNP core protein	www.genome.jp/kegg/pathway

Gene product names are common designations of the human and/or yeast orthologs.

a –TgRRM1^myc^ was immunoprecipitated (IP) from whole cell lysates (WCL) or nuclear extracts (NE). Note ten protein hits were identified in both extracts.

b – Gene ID for each protein hit was obtained from www.toxodb.org.

c – mRNA abundance in the tachyzoite stage (as percentile) for each gene was obtained from www.toxodb.org.

d – Peak expression of the encoded mRNA in the G1 subtranscriptome is indicated (ref #13); n/p = constitutive expression (non-periodic).

e – Interaction of the human orthog with RBM42 as predicted in high throughput studies (www.ncbi.org); N/A = not applicable.

f – references are included for the known splicing subcomplex associations.

## Discussion

Combining chemical mutagenesis with forward genetics to identify regulators of the eukaryotic cell cycle was introduced more than 40 years ago [19], [20] and remains a direct approach to uncover novel protein mechanisms affecting cell division. More than a decade ago we adapted this strategy to study unique mechanisms in *Toxoplasma* division, yet the impact of our studies is not restricted to apicomplexan cell cycles. Important proteins spanning evolution both recent and long conserved are emerging from the study of this collection of ts-mutants. Here we describe the discovery of a conserved protein (TgRRM1) that is required for cell cycle progression through an essential role in mRNA splicing. The chemical mutant that provided these insights rapidly arrests in the G1 phase when shifted just 6°C higher than a safe growth temperature (34°C) due to a point mutation in the TgRRM1 gene sequence. The TgRRM1 gene expression mechanism extends beyond the confines of apicomplexa biologic inventions as the human ortholog, RBM42 fully rescued the growth of this mutant and its splicing defect.

Splicing of mRNA is a fundamental process for gene expression in eukaryotes. Recognition of the splicing signals and removal of non-coding regions of pre-mRNA (introns) is carried out by a megadalton multi-subunit complex called the spliceosome. More than 150 proteins and five snRNAs (U1, U2, U4, U5, and U6) are required for a sequential assembly and activation of the spliceosome (for review see [21], [22], [23], [24]). Management of the complexity of spliceosome protein components is solved by pre-packing groups of proteins and RNA into building blocks that are detectable as stable snRNP particles (see Fig. 8B for diagram of spliceosome assembly). U1 snRNP particles are first recruited to 5′ splicing site (complex E), followed by the U2 snRNP association with the 3′ splicing site (complex A). It is known that assembly of spliceosome on the active splicing site requires the tri-snRNP that is formed from the U4/U6 di-snRNP and U5 snRNP. Together U1 and U2 snRNPs and the tri-snRNP form the pre-spliceosome complex B [24], [25]. Finally, several rearrangements within the complex B complete spliceosome activation (complex B*). The tri-snRNP complex contains a few unique components known to associate only with the assembled tri-snRNP particle prior to integration into the pre-splicesome such as SAD1 and SART1 [26]. Unlike U1 and U2 snRNPs, the tri-snRNP complex also undergoes dramatic rearrangements during each splicing cycle. U4 and U6 RNAs undergo re-annealing and modification before re-assembly into the U4/U6 di-snRNP. This renewed complex in turn is integrated into a functional tri-snRNP complex. We identified many components of the tri-snRNP complex, but not U1 or U2 snRNPs in TgRRM1 pull-downs including the two tri-snRNP-specific factors, the ortholog of hSAD1 (TGME49_094360) and hSART1 (TGME49_118140). These results indicate TgRRM1 has a role in formation of the pre-splicesome complex B particle that is downstream of assembly of the U4/U6 and U5 snRNPs particles. This role for TgRRM1 could also involve post-spliceosome dissassembly and recycling of components into new active spliceosomes. Recent reports demonstrate PRP4 kinase has a role in tri-snRNP formation through phosphorylation of PRP6 and PRP31 [27] with the loss of the PRP4 kinase activation step leading simultaneously to defects in mRNA splicing and specific cell cycle arrest at G1/S and G2/M transitions [27], [28]. In our TgRRM1 pull-down experiments, we also detected both of these factors necessary for tri-snRNP formation; U5 snRNP-specific PRP6 (TGME49_005220) and U4/U6 snRNP-specific PRP31 (TGME49_044100). Importantly, defects in TgRRM1 leads to a specific cell cycle arrest similar to what occurs in yeast PRP4 kinase mutants [28] with the primary block in *Toxoplasma* at the G1 to S phase transition, which may be related to the absence of a G2 period in the parasite asexual cell cycle [6], [7]. It is intriguing to speculate that the essentiality of TgRRM1 is related to the limiting step of tri-snRNP assembly thought to link spliceosome formation to cell cycle progression. Although compositions of the specific snRNPs and spliceosomal complexes (E, A, B and others) are thought to be well established, new factors are occasionally reported in various proteomics studies revealing the likelihood there is greater complexity in the splicing machinery than currently outlined in reviews of mRNA splicing [29], [30], [31]. This study presents a new essential protein that associates with tri-snRNP in *Toxoplasma* tachyzoites as a case in point. Whether TgRRM1 should be considered an integral component of the U4/U6.U5 tri-snRNP complex or a transient regulator of complex assembly will require further dissection of the mechanism involved. Interestingly, data mining of proteomics studies in human and *Drosophila melanogaster* reveal a transient and selective association of the TgRRM1/RBM42 orthologs with spliceosome complex B [32], [33]; the impact of this interaction on cellular mechanisms was not determined. Here the discovery of a role for TgRRM1 in the assembly of the spliceosome opens a new chapter in understanding how this protein family regulates mRNA splicing.

The ultimate goal of cells in G1 phase is to prepare for the next round of cell division, which a cell commits to once chromosome synthesis begins in S phase. The G1 phase is the most variable, and often the longest phase of the cell cycle, and appears to be well conserved in evolution. Such universal steps as first making proteins needed for gene expression such as transcription, splicing, and translation factors followed by building the key DNA synthetic factors appears to be an ancient G1 order [13], [34], [35], [36]. Despite the functional conservation of G1, the regulatory network controlling G1 progression is not necessarily shared. We noted previously [13] the traditional order of gene expression unfolding in the G1 phase of *Toxoplasma* tachyzoites was not governed by the retinoblastoma/E2F transcriptional network of higher eukaryotes [37] or by the alternative yeast G1 network involving SBF/MBF transcription complexes [38] as all these proteins are absent in the Apicomplexa. If checkpoint G1 regulators differ between eukaryotes, a more fundamental mechanism must be responsible for the traditional biosynthetic progression in G1. In this study we have discovered an essential splicing factor conserved across a billion years of evolution whose function is required for G1 progression into S phase in Apicomplexans. It is well documented that splicing and transcription are intimately connected [22], [39], [40] and there is a heavy dependence on gene expression in the G1 period (50% more mRNAs peak in G1 than S/M in *Toxoplasma*) [13]. Nearly all the splicing factors interacting with TgRRM1 follow the G1 peak expression timing of TgRRM1 itself (see Table 1). This timing is not coincidental as transcription and splicing activity is not equal across the cell division cycle in higher eukaryotes. In the open mitosis of animal cells, transcription is repressed from prophase to early telophase, at which time various splicing components appear to be held inactive in discrete compartments [39]. Transcription recommences in the late telophase with reassembly of the nuclear envelope and this is accompanied by the import of the splicing machinery stored in these compartments. Some details of this mechanism do not apply to *Toxoplasma* where chromosome segregation is endomitotic. However, we can not rule out the export/import of key splicing factors and there is a significant downregulation of mRNAs during mitosis/cytokinesis [13] providing evidence for temporal regulation of transcription/splicing in the *Toxoplasma* cell cycle. The results shown here suggest there is another simple model for regulating cell cycle progression through the strict timing of key splicing regulators like TgRRM1. In the absence of other cell cycle network controls [6], [7], [41], the “just-in-time” delivery of essential proteins appears to be a dominant method for regulating outcomes in Apicomplexa replication [14]. Thus, it is possible that basic timing mechanisms that achieve sufficient coordinate control in the ancient Apicomplexa may have since been modified to more nuanced and complex strategies in higher eukaryotes or were lost when the dependent mechanism was reduced such as in splicing of yeast mRNAs that lacks any ortholog of TgRRM1.

## Materials and Methods

### Cell culture

Parasites were grown in human foreskin fibroblasts (HFF) as described [42]. All transgenic and mutant parasite lines are derivatives of the RHΔ*hxgprt* parasite strain. Temperature sensitive clone 12-109C6 was obtained by chemical mutagenesis of the RHΔ*hxgprt* strain [8]. Growth measurements were obtained using parasites pre-synchronized by limited invasion as previously described [10], [43]. Vacuoles in the infected plates were evaluated over various time periods with average vacuole sizes determined at each time point from 50–100 randomly selected vacuoles. *Plasmodium falciparum* NF54 parasites were cultured at 37°C in 5% hematocrit (O-positive blood) RPMI1640 (Life Technologies), 0.5% Albumax, or 10% human AB serum.

### Immunofluorescence and flow cytometry

Confluent HFF cultures on the glass coverslips were infected with parasites for the indicated time. Cells were fixed in 3.7% paraformaldehyde, permeabilized in 0.25% Triton X-100 and blocked in 1% BSA in PBS. Incubations with primary antibody (1 h) followed by the corresponding secondary antibody (1 h) were performed at room temperature with DAPI (0.5 µg/ml) added in the final incubation to stain genomic DNA. The following primary antibodies were used at the indicated dilutions: mouse monoclonal anti-myc (Santa Cruz Biotechnology, Santa Cruz, CA), anti-centrin 26-14.1 (kindly provided by Dr. Jeffrey Salisbury, Mayo Clinic, Rochester, NY) and anti-IMC1 (kindly provided by Dr. Gary Ward, University of Vermont, VT) at 1∶1000. Serum raised against the conserved human centrin 1 (26-14.1) was previously shown to cross-react with the *Toxoplasma* centrin ortholog [10], [44]. All Alexa-conjugated secondary antibodies (Molecular Probes, Life Technologies) were used at dilution 1∶1000. After several washes with PBS, coverslips were mounted with Aquamount (Thermo Scientific), dried overnight at 4°C, and viewed on Zeiss Axiovert Microscope equipped with 100× objective. Images were processed in Adobe Photoshop CS v4.0 using linear adjustment for all channels. *P. falciparum* NF54 parasites were synchronized two times, 8 hours apart, using 5% sorbitol, in two continuous intraerythrocytic cycles. Immunofluorescence assays were performed as described before [45]. In brief, parasite cultures from ring (8–16 hours post-invasion), trophozoite (24–32 hours post-invasion), and schizont (36–44 hours post-invasion) stages were fixed overnight in 4% paraformaldehyde and 0.0075% glutaraldehyde in RPMI medium, permeabilized in 0.1% Triton X-100 in PBS, blocked in 3% bovine serum albumin (BSA), incubated with a 1∶100 dilution of anti-PfRRM1 antibody, probed with a 1∶70 dilution of FITC-labeled goat anti-rabbit secondary antibody (KPL) and 10 µg/ml Hoechst 33342 (Life Technologies), and visualized by microscopy.

Nuclear DNA content of mutant 12-109C6 parasites was evaluated by flow cytometry using SYTOX Green (Life Technologies) staining of tachyzoites as previously described [41]. Briefly, purified parasites were fixed in 70% ethanol and incubated at −20°C for at least 24 h. Fixed cells were stained with 1 µM SYTOX Green in 50 µM Tris pH 7.5 and treated with RNase cocktail (250 U; dark, room temperature) at a final concentration of 6×10^6^ parasites/ml. Nuclear DNA content was measured based on fluorescence (FL-1) using a 488 nm argon laser flow cytometer. Fluorescence was collected in linear mode (10,000 events) and the results were quantified using CELLQuest v3.0 (Becton-Dickinson Inc.).

### Genetic rescue and secondary complementation

Mutant 12-109C6 was complemented using the ToxoSuperCos cosmid genomic library as previously described [8], [10]. Briefly, mutant parasites were transfected with cosmid library DNA (50 µg DNA/5×10^7^ parasites/transfection) in twenty independent electroporations. After two consecutive selections at 40°C and than combination of high temperature and 1 µM pyrimethamine, double resistant populations were passed four times before genomic DNA was collected. Cosmid tags were recovered from the genomic DNA by plasmid-rescue protocols [8]. To identify the rescue locus rescued genomic inserts were sequenced using a T3 primer and the sequences mapped to the *Toxoplasma* genome (ToxoDB: http://www.toxodb.org/toxo/).

To resolve the contribution of individual genes in the locus, Gateway-based entry clones (Life Technologies) were build for three ORFs: TGME49_003100 (predicted by ToxoDB, ver.4.0, but was not confirmed by later versions), TGGT1_017850 and TGGT1_017860. Each construct included predicted genomic coding region and 1 kb genomic sequence including 5′UTR and 3′UTR regions. Cloning primers with incorporated attB-recombination sites are listed in the Dataset S1. Constructs were electroporated (5 µg DNA/5×10^7^ parasites) in the mutant 12-109C6 and parasite survival was monitored at 40°C.

### Structure-function studies

All ectopic construct designs and primers are listed in Dataset S1. The *wt-* and *ts-*alleles of TGGT1_017860 were cloned into pDEST_gra-myc_3X_/sag-HXGPRT vector, which provides a C-terminal myc_3X_ tag. Genomic locus including 2 kb of the promoter region was amplified from genomic DNA purified from parent RHΔ*hxgprt* strain and the mutant 12-109C6 using TGGT1_017860_FOR_attB1 and TGGT1_017860_REV_attB2 primers (Dataset S1). Plasmids were electroporated in the mutant 12-109C6 and selected on the medium with mycophenolic acid and xanthine. Stable clones were established and tested for growth at 40°C.

To perform a structure-functional analysis of TgRRM1, constructs expressing either a full-length protein or its subdomains were designed. PCR products were obtained by amplification from the RHΔ*hxgprt* cDNA library using the following set of primers listed in Dataset S1: TGGT1_017860_FOR_MfeI and TGGT1_017860_REV_SbfI (wt); TGGT1_017860_FOR_N_MfeI and TGGT1_017860_REV_SbfI (ΔN); TGGT1_017860_FOR_MfeI and TGGT1_017860_REV_Cc_SbfI (ΔCc); TGGT1_017860_FOR_N_MfeI and TGGT1_017860_REV_Cc_SbfI (ΔNCc); TGGT1_017860_FOR_MfeI and TGGT1_017860_REV_Ca_SbfI (ΔCa); TGGT1_017860_FOR_N_MfeI and TGGT1_017860_REV_Ca_SbfI (ΔNCa). Human RBM42 (96–1440 bp of the coding sequence) was amplified from the cDNA library of HFF cells with RBM42_FOR_MfeI and RBM42_REV_SbfI primers. PCR products were cloned into expression vector ptub- DD^L106P^-myc_3X_/sag-CAT using unique MfeI and SbfI cloning sites. The expression constructs had FKBP destabilizing domain (DD^L106P^) [46] and three copies of the myc epitope tag fused to the N-terminus of the polypeptide in an expression context driven by α-tubulin promoter. Chimeric Tg/PfRRM1 protein was designed in a way that the full sequence of PfRRM1 was flanked with the first 76 residues of TgRRM1 at the N-terminus and the last 45 residues of TgRRM1 at the C-terminus (see Figure S4). Recombinant cDNA of the chimeric protein was synthesized and cloned into ptub- DD^L106P^-myc_3X_/sag-CAT vector by GenScript (GenScript USA). Plasmid constructs were transfected in the mutant 12-109C6 and stable transgenic clones were selected with 20 µM chloramphenicol. Presence of the ligand shield 1 stabilized the RRM1 recombinant proteins with DD-domain, however, basal expression from the tubulin promoter (without shield 1) slightly exceeded the endogenous levels of TgRRM1 (data not shown), therefore, shield 1 was not included in any complementation assays and was only used in immunofluorescent analysis to facilitate protein visualization.

### Antibody production and Western blot analysis

PfRRM1 coding sequence was amplified from the cDNA library of the blood stage cells of *Plasmodium falciparum* NF54 strain using primers PF13_0318_FOR_GST_SmaI and PF13_0318_REV_GST_NotI (Dataset S1). PCR fragment was cloned into expression vector pGEX6T-2 (GE Healthcare) that introduced a Glutathione-S-transferase (GST) tag to the N-terminus of the protein. Recombinant GST-PfRRM1 was expressed in bacteria, purified on Gluthatione-agarose beads (Sigma-Aldrich), and used to immunize rabbits. Anti-PfRRM1 antibodies were affinity-purified from the serum using nitrocellulose strips with electroblotted PfRRM1 protein. Purified parasites were washed in PBS and collected by centrifugation. Total lysates were obtained by mixing with Leammli loading dye, heated at 95°C for 10 min, and briefly sonicated. After separation on the SDS-PAGE gels proteins were transferred onto nitrocellulose membrane and probed with anti-PfRRM1 antiserum. After incubation with secondary HRP-conjugated anti-rabbit antibody, proteins were visualized in enhanced chemiluminescence reaction. Confirmation of the PfRRM1 rabbit antiserum specificity is shown in Figure S2. To evaluate PfRRM1 in merozoites, twenty million *P. falciparum* NF54 parasites each from synchronized ring, trophozoite, and schizont stages were treated with 0.1% saponin (Sigma-Aldrich), washed in PBS, resuspended directly in equal volumes of 2× SDS-PAGE sample buffer (Bio-Rad), boiled for 5 min and used for Western analysis. Anti-Histone H3 antibody (Abcam) was used as a loading control.

### Identification of TgRRM1 orthologs

Orthologs of TgRRM1 (TGGT1_017860) were identified by BLAST and aligned by ClustalW2. Analyzed species and corresponding gene ID: *Toxoplasma gondii* (TGGT1_017860), *Neospora caninum* (NCLIV_021840), *Babesia bovis* (BBOV_II004860), *Theileria annulata* (TA12210), *Eimeria tenella* (ETH_00023710), *Cryptosporidium parvum* (cgd1_1070), *Plasmodium falciparum* (PF13_0318), *Plasmodium vivax* (PVX_114975), *Perkinsus marinus* (XP_002778935.1), *Paramecium tetraurelia* (XP_001433574.1), *Caenorhabditis elegans* (NP_498090.1), *Drosophila melanogaster* (NP_649552.1), *Arabidopsis thaliana* (NP_187100.1), *Mus musculus* (NP_598454.2), *Homo sapiens* (NP_077297.2). Protein alignments are shown in Figure S3.

### RNA isolation and microarray analysis

RNA was extracted from parasites using the RNeasy kit with β-mercaptoethanol and DNase I treatment (Qiagen). RNA quality was determined using the Agilent Bioanalyzer 2100 (Santa Clara, CA). A total of 500 ng starting RNA was used to produce cRNA using the Affymetrix One-Cycle Kit (Affymetrix, Santa Clara CA). Fragmented cRNA (5 µg) was hybridized to the *Toxoplasma gondii* Affymetrix microarray according to standard hybridization protocols (ToxoGeneChip: http://ancillary.toxodb.org/docs/Array-Tutorial.html). Two hybridizations were done for each sample type and all data were deposited at NCBI GEO (GSE43315). Hybridization data was preprocessed with Robust Multi-array Average (RMA) and normalized using per chip and per gene median polishing and analyzed using the software package GeneSpring GX (Agilent Technologies, Santa Clara CA). An ANOVA or t-test were run in order to identify genes with significantly greater than random variation in RNA abundance across the data grouped by either temperature or mutant type. Variances were calculated using cross-gene error model, with a p-value cutoff 0.05, and multiple testing correction: Benjamini and Hochberg False Discovery Rate. This restriction tested 8,131 probe sets.

### Deep RNA sequencing

Ambion MicroPoly(A)Purist Kit (Ambion) was used for enrichment of transcripts. The SOLiD Total RNA-Seq Kit (Life Technologies) was used to construct template cDNA for RNA-Seq following the protocol recommended by Applied Biosystems (Life Technologies). Briefly, mRNA was fragmented using chemical hydrolysis followed by ligation with strand specific adapters and reverse transcript to generate cDNA. The cDNA xfragments, 150 to 250 bp, were subsequently isolated by electrophoresis in 6% Urea-TBE acrylamide gel. The isolated cDNA was amplified through 15 amplification cycles to produce the required number of templates for the SOLiD EZ Bead system (Life Technologies), which was used to generate template bead library for the ligation base sequencing by the SOLiD 4 instrument (Life Technologies).

Mapping of SOLiD reads was analyzed using the Whole Transcriptome analysis pipeline in the Applied Biosystems BioScope software (Life Technologies). Mapping was done twice for each sample, once against the genome of *T. gondii* strain ME49, and a second time against the genome of *T. gondii* strain GT1. Fasta files and corresponding GFF files (converted to GTF files) were obtained for both reference genomes from the ToxoDB web site (www.toxodb.org; Release 6.1). BioScope parameter settings were left at the default mapping values. The filter file used consisted of the short adapter sequences. Each pipeline run generated an alignment report and a filtering report. The read counts were summed in a set of *.wig files (two for each chromosome, corresponding to the strands) and in an exon rollup file that summed to the exon/gene level based on the coding region locations given in the GTF file. Finally, three BAM files (binary compressed versions of Sequence Alignment/Map (SAM) files) were created that stored the mapping information for each individual read: one for mapped reads, one for unmapped reads, and one for the filtered reads. An index file was created for the BAM file, storing the location of the mapped reads. Note: the Fasta files from ToxoDB had, in addition to Fasta entries for the 14 chromosomes in *T. gondii*, Fasta entries for several hundred short floating contigs that could not be placed on a chromosome (308 contigs in ME49 strain, 351 such in GT1 strain). For use in BioScope, which puts a limit on the number of separate Fasta sequences to which reads can be mapped, these large sets of floating contigs were combined into an artificial 15^th^ chromosome, each contig separated by a set of 60 N's from neighboring contigs. With our SOLiD reads being a maximum of 50 bases long, an interval of 60 N's clearly separated the contigs for mapping use.

For each gene, the total number of RNA reads aligning to either exons or introns using the “coverageBed” program was calculated, which is part of the BEDTools suite [47]. In order to estimate mRNA expression levels from the RNA sequencing data (RPKM normalized according to [48]), we determined the number of reads that aligned to the predicted exons for each gene model excluding hits to predicted intron regions (ME49 strain, ToxoDB release 6.1). For those genes predicted to have introns, we calculated the ratio of intronic to exonic reads (I/E). The I/E ratio quantifies the relative abundance of unspliced versus spliced transcripts for each gene. Mature transcripts will have I/E values close to zero, while unspliced message will have relatively higher I/E values. Genomic coordinates of exons were obtained from ToxoDB (ME49 strain, release 6.1) and derived intron positions were based on the exon coordinates. If there is no splicing defect, the ratio of I/E values for a particular gene between conditions were expected to be equal (I/E_y_ = I/E_x_). To conservatively measure the extent to which two I/E ratios differ, the shortest line between the point (I/E_x_,I/E_y_) and the diagonal line (y = x) was calculated and this value was designated the “distance” between the I/E ratios in Equation 1. A two-tailed Student's t-test was performed on the distances for each RNA-sequencing experiment in order to test for significant changes between mRNA splicing under different conditions.
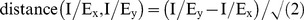
(1)


### Cell lysis and immunoprecipitation

For proteomics studies parasites were grown at 34°C for 48 hours. Infected monolayers of HFF cells were washed once with PBS and collected by centrifugation at 4°C for 10 min at 700×*g*. Cell resuspended in cold PBS were passed sequentially through 20/23/25-guage needles to release parasites from host cells and parasitophosphorous vacuoles. Parasites were pelleted (4°C for 15 min at 700×*g*) and counted. Whole cell lysates were obtained by lysing 2×10^9^ parasites for 60 min at 4°C in the lysis buffer (0.1% [v/v] Nonidet P-40, 10 mM HEPES pH 7.4, 150 mM KCl, with protease inhibitors), followed by five cycles of snap freezing in the liquid nitrogen bath and slow thawing in ice-water bath. Lysates were clarified by centrifugation at 12,000×*g* for 30 minutes at 4°C. To make nuclear extracts, 2×10^9^ parasites were first lysed for 5 min on ice in the lysis buffer A (0.1% [v/v] Nonidet P-40, 10 mM HEPES pH 7.4, 10 mM KCl, 10% [v/v] glycerol, with protease inhibitors), then centrifuged at 6,000×*g* for 8 min at 4°C. Nuclei pellet was further lysed at 4°C in lysis buffer B (0.1% [v/v] Nonidet P-40, 10 mM HEPES pH 7.4, 400 mM KCl, 10% [v/v] glycerol with protease inhibitors), and subjected to five cycles of freezing-thawing. Nuclear extracts were clarified by centrifugation at 12,000×*g* for 30 minutes at 4°C. Protein extracts were rotated overnight at 4°C with magnetic beads (MBL International, MA) containing pre-bound monoclonal anti-myc antibody (Santa Cruz Biotechnology, Santa Cruz, CA). After five washes with cold lysis buffer, bound proteins were eluted in 50 µl Laemmli sample buffer for 5 min at 95°C. Precipitated complexes were separated by SDS-PAGE (Any kD precast polyacrylamide gel; Bio-Rad) and stained with Coomassie Blue (GelCode Blue Stain Reagent, Pierce). The entire length of each sample lane was cut into 24 slices that were maintained in MilliQ water for mass-spectrometry.

### Liquid chromatography coupled to mass spectrometry (LC/MS-MS)

Proteins from a coomassie-stained gel slice were reduced and alkylated with TCEP and iodoacetamide prior to trypsin digest (10 ng/µl in 25 mM ammonium bicarbonate, 0.1%ProteaseMax (Promega)) for 1 hour at 50°C. Nanospray LC-MS/MS was performed on a LTQ linear ion trap mass spectrometer (LTQ, Thermo, San Jose, CA) interfaced with a Rapid Separation LC 3000 system (Dionex Corporation, Sunnyvale, CA). Samples were run sequentially on Acclaim PepMap C18 Nanotrap column (5 µm, 100 Å,/100 µm i.d. ×2 cm) followed by Acclaim PepMap RSLC C18 column (2 µm, 100 Å, 75 µm i.d. ×25 cm) (Dionex Corp). Peptides eluted with gradient mobile phase A (2%Acetonitrile/water +0.1% formic acid) and mobile phase B (80% acetonitrile/water +0.1% formic acid) were assessed for MS/MS analysis. Raw LC-MS/MS data was collected using Proteome Discoverer 1.2 (Thermo Scientific). Created mgf files were used to search the Toxo_Human Combined database using in-house Mascot Protein Search engine (Matrix Science). Final list was generated by Scaffold 3.5.1 (Proteome Software) with following filters: 99% minimum protein probability, minimum number peptides of 2 and 95% peptide probability.

## Supporting Information

Dataset S1 Primers designed and transgenic clones generated by this study. The primers worksheet includes names, nucleotide sequence and comments for each primer used in the study. The transgenic strains worksheet lists plasmids constructs, the source of parental *Toxoplasma* strains and drug selection used to obtain each transgenic clone.
(XLSX)

Dataset S2 Microarray analysis of mutant 12-109C6. The list of genes with statistically different mRNA (anova test) expression in the mutant 12-109C6 grown at 34°C versus 40°C. For details of the experiment and analysis see Material and Methods and GEO dataset GSE43315 (ncbi.org). Data entry in this worksheet includes Genebank ID (ToxoDB.org), fold difference of expression (FC), RMA values from current microarray. Data was categorized (Category) based on the gene product description (Description) and mRNAs predicted to peak in G1 (G1 sub), S/M (S/M sub) and non-cyclical (not periodic) are indicated with x.
(XLSX)

Dataset S3 Analysis of splicing defect of 12-109C6 based on deep RNA sequencing. The worksheet includes Genebank ID (ToxoDB.org), results of intronic over exonic hits (ie) quantifications described in Matherial and Methods and gene product description (Description). Cyclical mRNAs are indicated with x (G1 sub and S/M sub).
(XLS)

Dataset S4 Proteomic analysis of immunoprecipitated TgRRM1 complex. Details of these experiments and mass-spectrometry analysis can be found in Material and Methods. Mass-spectrometry results of immunoprecipitated complexes from whole cell lysate (WCL, blue border) or nuclear extracts (NE, pink border) are shown. Protein identified in the negative control (mutant 12-109C6) is highlighted in grey. The worksheet contains data entry for Genebank ID (ToxoDB.org), gene product description, molecular weight of the protein in Da, protein identification probability (ID probability), number of unique peptides and unique spectra, number and percentage of unique spectra, and percentage of sequence coverage for each identified protein.
(XLSX)

Figure S1 Subdomain structure of the RNA recognition motif of TgRRM1 protein. (A) Conserved RNA-binding sequences RNP1 and RNP2 are enclosed in the grey boxes. Aromatic residues involved in the primary binding of RNA are shown in bold and enlarged. Tyrosine 169 mutation to asparagine marked with an asterisk. Prediction of the folding was generated using Jpred3 software (http://www.compbio.dundee.ac.uk/www-jpred/). Beta-folds (block arrows) and alpha-helical structures (boxes) are labeled and numbered [51]. (B) Predicted tertiary structure of the RRM domain of TgRRM1 protein. Location of the RRM domain in the protein sequence is indicated with a grey box. RRM domain (123–206 residues) was modeled into the template 2dgoA (http://swissmodel.expasy.org/). A typical fold of four beta-sheet packed against two alpha-helixes are shown in two views. (C) Predicted organization of structured and unstructured domains of TgRRM1 (red), PfRRM1 (green) and human RBM42 (blue). The order/disorder plot was generated using PONDR prediction algorithm (http://www.pondr.com/). All proteins were aligned relative to the position of RRM domain shown as a grey box on the top of the graph. The three orthologs show a similar folding pattern in the areas surrounding the RRM domain. Arrows point toward identical pattern of the downhill slop, which was identified as a positively charged area, required for TgRRM1 nuclear retention (see Figure 5).
(TIF)

Figure S2 Anti-PfRRM1 serum analysis. Polyclonal rabbit antiserum raised against recombinant PfRRM1 cross-reacts with TgRRM1. Western blot analysis of asynchronous populations of the blood stage *P.falciparum* NF54 parasites (lane Pf) and mutant 12-109C6 parasites (lanes Tg) grown at 34°C and 40°C. Total lysates (equivalent of 10^7^ parasites) were separated on 10% SDS-PAGE, transferred to nitrocellulose membrane and probed with the new anti-PfRRM1 antiserum. A single major band with the correct predicted molecular weight (PfRRM1 – 21 kDa; TgRRM1 – 33 kDa) was detected in either species. Note also that when grown at the restricted temperature, TgRRM1 in mutant 12-109C6 parasites was undetectable in whole cell lysates consistent with the instability of the ts-TgRRM1 isoform in Figure 4. A star indicates a faint band present only in *Toxoplasma* samples, which is likely a result of HFF host cell contamination. Interestingly, this band migrates according to the predicted size of human ortholog RBM42 (50 kDa). Molecular mass standards are indicated to the left.
(TIF)

Figure S3 Alignment of TgRRM1 orthologs. Alignment of the protein sequences of the RRM1 orthologs from different organisms was build using ClustalW2 software (http://www.ebi.ac.uk/Tools/msa/clustalw2/). RRM domain (solid line) and positively charged region implicated in the nuclear targeting (dotted line) are outlined. Star indicates a conservative position, which is mutated in tsTgRRM1. Grey shading highlights identical/conservative residues.
(TIF)

Figure S4 Designs for the chimera Tg/Pf RRM1 protein.
(DOCX)

Figure S5 Steady state mRNA levels are dramatically affected in mutant 12-109C6. (A) Box-and-whisker plot of the distances (equation 1) between RPKM normalized RNA levels comparing the mutant 12-109C6 parasites grown at 34°C versus 40°C (green) or mutant parasites at 34°C versus complemented parasites at 40°C (blue). Distance values close to zero indicate minimal changes between samples in detected steady state RNA levels. (B) RPKM normalized mRNA levels of single exon genes. The x-axis represents the expression levels of genes in the mutant at 34°C and the y-axis plots levels of the mutant grown at 40°C. The blue line is y = x and the expected value if there is no change in gene expression between samples. Both axes are plotted in log scale. (C) RPKM normalized mRNA levels of the complemented strain (wt-TgRRM1 allele) at 40°C on the y-axis versus the mRNA levels of the mutant parasites grown 34°C.
(TIF)

Table S1 RRM domain-containing proteins of *T. gondii*. *Toxoplasma* genome has 86 proteins that contain one or more RRM domains. 50 RRM proteins have orthologs in *Plasmodium falciparum*. Data analysis performed in ToxoDB, PlasmoDB and ncbi. Conservative proteins with known function were identified in BLAST search (ncbi.org). Orthologs had e-values −10 or lower. a – domains were identified according to ncbi.org. * - more than one *Toxoplasma* gene has the same orthologous protein in *Plasmodium falciparum*.
(DOCX)

Table S2 Mutation in TgRRM1 leads to global mRNA splicing defect.
(DOCX)
