# Supplementary figures and images for: Discovery of a Splicing Regulator Required for Cell Cycle Progression
Source: PLoS Genet. 2013 Feb 21;9(2):e1003305. doi: 10.1371/journal.pgen.1003305 (PMC3578776; doi:10.1371/journal.pgen.1003305)

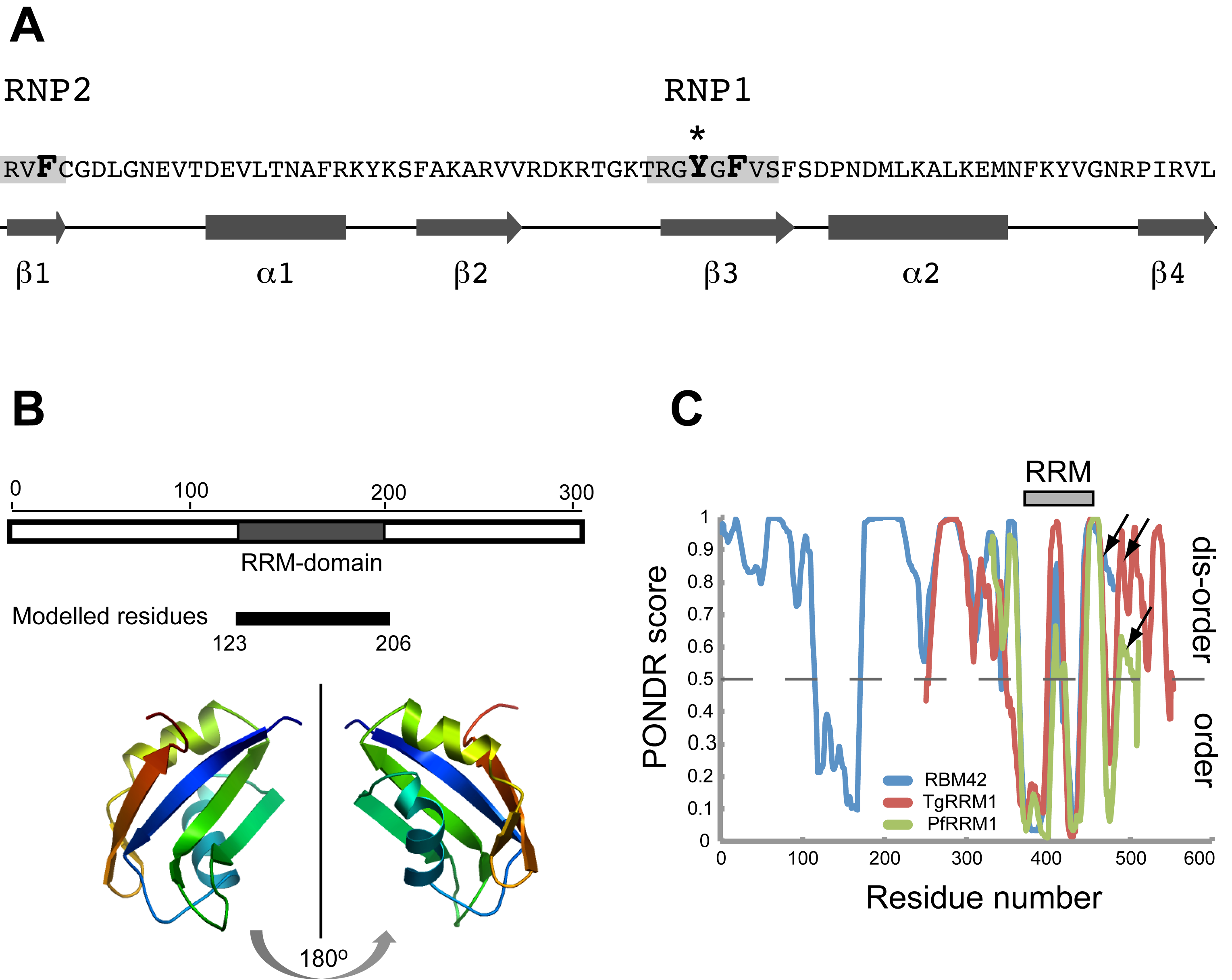

Supplement: Figure S1 — Subdomain structure of the RNA recognition motif of TgRRM1 protein. (A) Conserved RNA-binding sequences RNP1 and RNP2 are enclosed in the grey boxes. Aromatic residues involved in the primary binding of RNA are shown in bold and enlarged. Tyrosine 169 mutation to asparagine marked with an asterisk. Prediction of the folding was generated using Jpred3 software (http://www.compbio.dundee.ac.uk/www-jpred/). Beta-folds (block arrows) and alpha-helical structures (boxes) are labeled and numbered [51]. (B) Predicted tertiary structure of the RRM domain of TgRRM1 protein. Location of the RRM domain in the protein sequence is indicated with a grey box. RRM domain (123–206 residues) was modeled into the template 2dgoA (http://swissmodel.expasy.org/). A typical fold of four beta-sheet packed against two alpha-helixes are shown in two views. (C) Predicted organization of structured and unstructured domains of TgRRM1 (red), PfRRM1 (green) and human RBM42 (blue). The order/disorder plot was generated using PONDR prediction algorithm (http://www.pondr.com/). All proteins were aligned relative to the position of RRM domain shown as a grey box on the top of the graph. The three orthologs show a similar folding pattern in the areas surrounding the RRM domain. Arrows point toward identical pattern of the downhill slop, which was identified as a positively charged area, required for TgRRM1 nuclear retention (see Figure 5). (TIF) [file pgen.1003305.s005.tif]

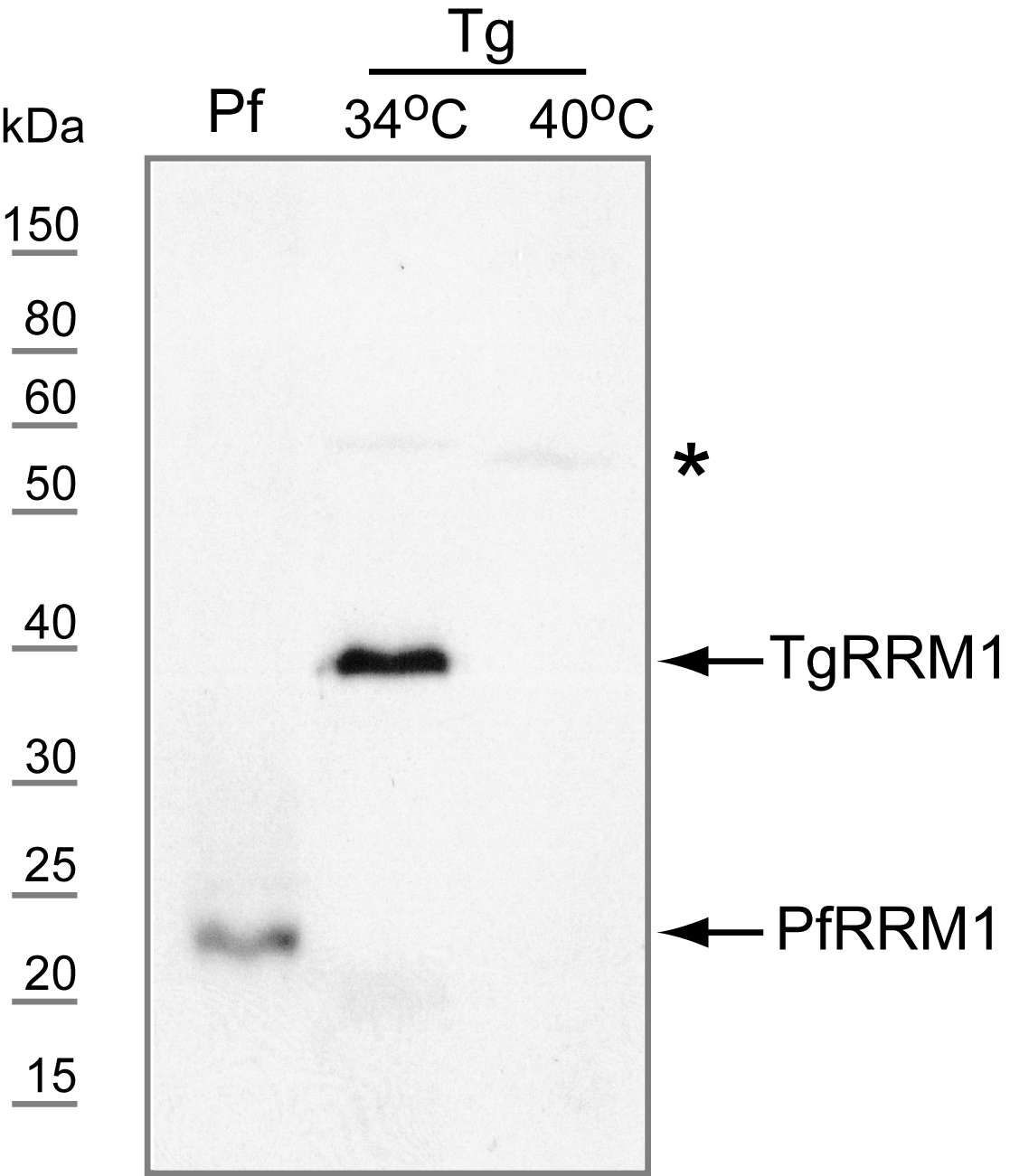

Supplement: Figure S2 — Anti-PfRRM1 serum analysis. Polyclonal rabbit antiserum raised against recombinant PfRRM1 cross-reacts with TgRRM1. Western blot analysis of asynchronous populations of the blood stage P.falciparum NF54 parasites (lane Pf) and mutant 12-109C6 parasites (lanes Tg) grown at 34°C and 40°C. Total lysates (equivalent of 107 parasites) were separated on 10% SDS-PAGE, transferred to nitrocellulose membrane and probed with the new anti-PfRRM1 antiserum. A single major band with the correct predicted molecular weight (PfRRM1 – 21 kDa; TgRRM1 – 33 kDa) was detected in either species. Note also that when grown at the restricted temperature, TgRRM1 in mutant 12-109C6 parasites was undetectable in whole cell lysates consistent with the instability of the ts-TgRRM1 isoform in Figure 4. A star indicates a faint band present only in Toxoplasma samples, which is likely a result of HFF host cell contamination. Interestingly, this band migrates according to the predicted size of human ortholog RBM42 (50 kDa). Molecular mass standards are indicated to the left. (TIF) [file pgen.1003305.s006.tif]

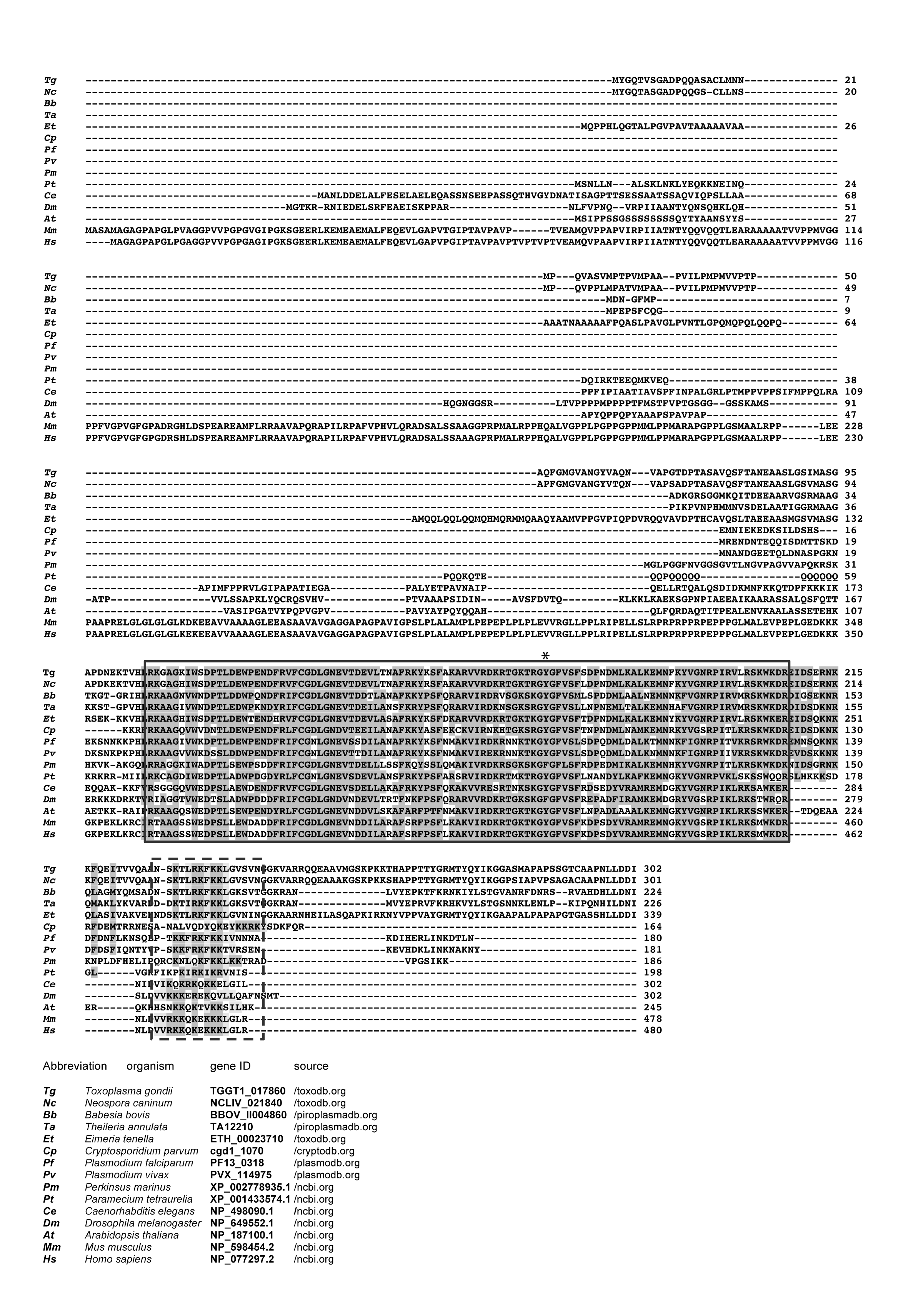

Supplement: Figure S3 — Alignment of TgRRM1 orthologs. Alignment of the protein sequences of the RRM1 orthologs from different organisms was build using ClustalW2 software (http://www.ebi.ac.uk/Tools/msa/clustalw2/). RRM domain (solid line) and positively charged region implicated in the nuclear targeting (dotted line) are outlined. Star indicates a conservative position, which is mutated in tsTgRRM1. Grey shading highlights identical/conservative residues. (TIF) [file pgen.1003305.s007.tif]

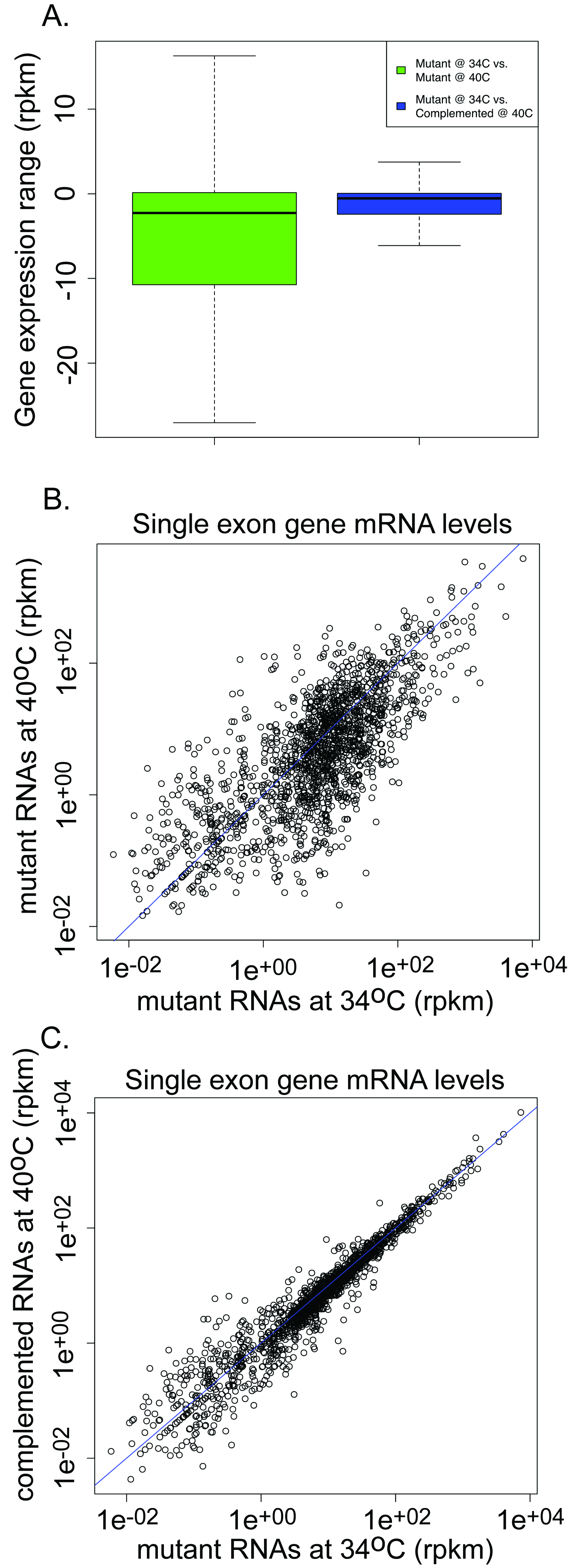

Supplement: Figure S5 — Steady state mRNA levels are dramatically affected in mutant 12-109C6. (A) Box-and-whisker plot of the distances (equation 1) between RPKM normalized RNA levels comparing the mutant 12-109C6 parasites grown at 34°C versus 40°C (green) or mutant parasites at 34°C versus complemented parasites at 40°C (blue). Distance values close to zero indicate minimal changes between samples in detected steady state RNA levels. (B) RPKM normalized mRNA levels of single exon genes. The x-axis represents the expression levels of genes in the mutant at 34°C and the y-axis plots levels of the mutant grown at 40°C. The blue line is y = x and the expected value if there is no change in gene expression between samples. Both axes are plotted in log scale. (C) RPKM normalized mRNA levels of the complemented strain (wt-TgRRM1 allele) at 40°C on the y-axis versus the mRNA levels of the mutant parasites grown 34°C. (TIF) [file pgen.1003305.s009.tif]
