# Supplementary material for: Discovery of a Splicing Regulator Required for Cell Cycle Progression
Source: PLoS Genet. 2013 Feb 21;9(2):e1003305. doi: 10.1371/journal.pgen.1003305 (PMC3578776; doi:10.1371/journal.pgen.1003305)
Supplement: Figure S4 — Designs for the chimera Tg/Pf RRM1 protein. (DOCX) [file pgen.1003305.s008.docx]

Suvorova Figure S4

Chimeric Tg/PfRRM1 nucleotide sequence

TgRRM1 (nucleotide positions 1 to 228)

**ATGTACGGACAGACTGTTTCGGGCGCTGACCCGCAGCAGGCGTCGGCCTGCCTAATGAATAATATGCCTCAGGTTGCATCCGTAATGCCCACGCCTGTCATGCCGGCGGCCCCCGTCATTCTGCCTATGCCTATGGTTGTTCCTACGCCAGCACAATTCGGCATGGGCGTTGCAAATGGATACGTAGCGCAGAATGTGGCACCAGGCACAGACCCCACGGCCTCGGCC**

PfRRM1 (nucleotide positions 1 to 540)

ATGAGGGAAAATGATAATACAGAACAACAAATATCAGATATGACAACTTCAAAGGATGAAAAATCTAATAACAAAAAGCCACATTTGAGAAAAGCAGCAGGTATTGTTTGGAAGGATCCTACATTAGACGAATGGCCAGAAAATGATTTTCGAATATTCTGTGGGAATTTAGGGAATGAAGTATCGAGTGATATTCTTGCTAATGCCTTTAGAAAATATAAGTCCTTTAATATGGCCAAGGTAATAAGAGATAAAAGAAATAATAAGACCAAAGGATATGGTTTCGTTTCTTTATCAGATCCTCAAGATATGTTAGATGCTTTAAAAACTATGAATAATAAATTTATAGGGAATAGACCTATAACCGTAAAAAGAAGTAGATGGAAAGATAGAGAAATGAATTCTCAGAAAAATAAAGACTTTGATAACTTTTTAAAAAATTCTCAATTACCTACAAAAAAATTTAGGAAATTTAAAAAAATCGTCAACAATAATGCTAAAGATATTCATGAAAGATTAATAAATAAAGATACCTTGAAC

TgRRM1 (nucleotide positions 1118 to 1256)

**TCGAAACCAAAGAAGACTCATGCGCCACCTACGACATACGGGCGAATGACCTATCAGTACATCAAAGGAGGCCCTTCCATGGCGCCTGCACCATCGTCCGGAACTTGTGCGGCACCGAACCTTTTGGATGACATATGA**

Chimeric Tg/PfRRM1 protein sequence

TgRRM1 (amino acid residues 1 to 76)

**MYGQTVSGADPQQASACLMNNMPQVASVMPTPVMPAAPVILPMPMVVPTPAQFGMGVANGYVAQNVAPGTDPTASA**

PfRRM1 (amino acid residues 1 to 180)

MRENDNTEQQISDMTTSKDEKSNNKKPHLRKAAGIVWKDPTLDEWPENDFRIFCGNLGNEVSSDILANAFRKYKSFNMAKVIRDKRNNKTKGYGFVSLSDPQDMLDALKTMNNKFIGNRPITVKRSRWKDREMNSQKNKDFDNFLKNSQLPTKKFRKFKKIVNNNAKDIHERLINKDTLN

TgRRM1 (amino acid residues 256 to 302)

**SKPKKTHAPPTTYGRMTYQYIKGGPSMAPAPSSGTCAAPNLLDDI***
