# Supplementary material for: Discovery of a Splicing Regulator Required for Cell Cycle Progression
Source: PLoS Genet. 2013 Feb 21;9(2):e1003305. doi: 10.1371/journal.pgen.1003305 (PMC3578776; doi:10.1371/journal.pgen.1003305)
Supplement: Table S1 — RRM domain-containing proteins of T. gondii. Toxoplasma genome has 86 proteins that contain one or more RRM domains. 50 RRM proteins have orthologs in Plasmodium falciparum. Data analysis performed in ToxoDB, PlasmoDB and ncbi. Conservative proteins with known function were identified in BLAST search (ncbi.org). Orthologs had e-values −10 or lower. a – domains were identified according to ncbi.org. * - more than one Toxoplasma gene has the same orthologous protein in Plasmodium falciparum. (DOCX) [file pgen.1003305.s010.docx]

**Table S1. RRM domain-containing proteins of *T.gondii***

| ***Toxoplasma* Gene ID** | **RRM ^a^** | **Other domains ^a^** | **Gene product description** | ***Plasmodium* gene ID** |
| --- | --- | --- | --- | --- |
| **Conserved RRM proteins with known function** | | | | |
| TGME49_031970 | 1 | Pro8NT, ProCN, PRP8_domainIV, MPN_PRP8, U5_2snR, U6_2snR | pre-mRNA splicing factor PRP8 | PFD0265w |
| TGME49_094670 | 1 | eIF3g | eukaryotic translation initiation factor 3 | MAL8P1.83 |
| TGME49_022860 | 1 | WD40 | eukaryotic translation initiation factor 3 subunit 9 | PFE0885w |
| TGME49_005180 | 1 | UsnRNP70_N | U1 small nuclear ribonucleoprotein | MAL13P1.338 |
| TGME49_027850 | 1 | Cyclophilin (2) | RNA recognition motif-containing protein | PF08_0086 |
| TGME49_036910 | 1 | zfCCCH | U2 snRNP auxiliary factor small subunit | PF11_0200 |
| TGME49_119850* | 1 | U2AF | U2 snRNP auxiliary factor subunit p65 | PF13_0147 |
| TGME49_114050* | 1 | U2AF | U2 snRNP auxiliary factor subunit p65 | PF13_0147 |
| TGME49_039410 | 1 | RI | CNOT4, CCR-NOT complex subunit | PFL1705w |
| TGME49_054650 | 1 |  | RBM22 | PFL2310w |
| TGME49_109800 | 1 |  | U1A small nuclear ribonucleoprotein | MAL13P1.35  PFI1695c |
| TGME49_068750 | 1 |  | peptidyl-prolyl cis-trans isomerase E | PF13_0122 |
| TGME49_011020 | 1 |  | embryonic poly(A)-binding protein 2 | PFI1175c |
| TGME49_070640 | 1 |  | SC35, SR-rich splicing factor | PF11_0279  PFE0160c |
| TGME49_089560 | 1 |  | nuclear cap-binding protein | PFD0750w |
| TGME49_083740 | 1 |  | 3'UTR mRNA cleavage stimulating protein | PFI1600w |
| TGME49_105010 | 1 |  | SF3b splicing complex subunit | PFL1200c |
| TGME49_064610* | 2 |  | hnRNP A1 like 2 | PFD0700c |
| TGME49_110050* | 2 |  | DAZ-associated protein 1 | PFD0700c |
| TGME49_024580 | 2 |  | SF3b splicing complex subunit 4 | PF14_0194 |
| TGME49_062620 | 2 |  | hnRNP M | PF10_0068 |
| TGME49_015230 | 2 |  | HIV-1 Tat specific factor 1 | MAL7P1.157a |
| TGME49_091930 | 2 |  | nucleolin | PFI0820c |
| TGME49_091950* | 2 |  | U2 snRNP auxiliary factor subunit p65 | PF14_0656 |
| TGME49_034520* | 3 |  | U2 snRNP auxiliary factor subunit p65 | PF14_0656 |
| TGME49_112530 | 3 |  | RBM23 | MAL13P1.120 |
| TGME49_108920 | 3 |  | U2 snRNP auxiliary factor | PF07_0066 |
| **RRM proteins with unknown function** | | | | |
| **Contain 3 or more RRM domains** | | | | |
| TGME49_118690 | 5 |  | RBM19 | PFL0830w |
| TGME49_120100 | 3 |  | RNA recognition motif-containing protein | MAL13P1.303-b |
| TGME49_036540* | 3 |  | RRM domain-containing protein | PF10_0235 |
| TGME49_066740 | 3 |  | DNA/RNA binding protein |  |
| TGME49_002770 | 3 |  | RNA-binding protein |  |
| TGME49_090660 | 3 |  | polypirimidine track-binding protein 3 | PFF0320c |
| TGME49_068200 | 3 | WW | RNA recognition motif domain-containing protein | PF13_0315  PF14_0096 |
| **Contain 2 RRM domains** | | | | |
| TGME49_105850 | 2 |  | RNA binding protein |  |
| TGME49_023930 | 2 |  | RNA binding protein |  |
| TGME49_121630 | 2 |  | hypothetical protein |  |
| TGME49_104760 | 2 |  | conserved hypothetical protein |  |
| TGME49_003530 | 2 |  | RNA recognition motif domain containing protein |  |
| TGME49_121360 | 2 | WW | RNA recognition motif domain-containing protein | MAL8P1.40 |
| TGME49_065530 | 2 |  | RNA binding motif-containing protein |  |
| TGME49_017540* | 2 |  | splicing factor | PFE0865c |
| TGME49_119530* | 2 |  | splicing factor | PFE0865c |
| TGME49_094710 | 2 |  | hypothetical protein |  |
| TGME49_068380 | 2 |  | RNA recognition motif-containing protein |  |
| TGME49_092200 and TGME49_092210 | 2 |  | poly(A) binding protein | PFI1025w |
| **Contain 1 RRM domain** | | | | |
| TGME49_067600 | 1 | DUF1768  FHA  SF-CC1 | RNA recognition motif-containing protein |  |
| TGME49_014820 | 1 | G-patch | DNA repair enzyme | PF14_0513 |
| TGME49_070770 | 1 | PWI | hypothetical protein | PFF0505c |
| TGME49_111070 | 1 | zinc finger (2) | hypothetical protein |  |
| TGME49_089010 | 1 | MPP_SW (2) CWf_J (2) | conserved hypothetical protein |  |
| TGME49_084050 | 1 | DEAH  DUF1605 | DEAH-box RNA/DNA helicase |  |
| TGME49_074140 | 1 | rad18 | RNA recognition motif-containing protein |  |
| TGME49_083880 | 1 | SF-CC1 | RBM34 | PF10_0194 |
| TGME49_006670 | 1 | SPOC | hypothetical protein |  |
| TGME49_040070 | 1 | WW | RNA recognition motif domain-containing protein | PFL1745c |
| TGME49_054220 | 1 |  | hypothetical protein |  |
| TGME49_021570 | 1 |  | hypothetical protein | PF07_0083 |
| TGME49_065250 | 1 |  | alpha-1 type II collagen |  |
| TGME49_054210 | 1 |  | hypothetical protein |  |
| TGME49_008350 | 1 |  | RNA recognition motif-containing protein |  |
| TGME49_012190 | 1 |  | RNA recognition motif domain containing protein |  |
| TGME49_091330 | 1 |  | hypothetical protein | PFF0760w |
| TGME49_011420 | 1 |  | hypothetical protein |  |
| TGME49_072440 | 1 |  | hypothetical protein |  |
| TGME49_094710 | 1 |  | hypothetical protein |  |
| TGME49_003540 | 1 |  | RNA binding protein |  |
| TGME49_003080 | 1 |  | RNA recognition motif-containing protein | PF13_0318 |
| TGME49_106600 | 1 |  | RNA recognition motif domain-containing protein |  |
| TGME49_015400 | 1 |  | RNA binding protein |  |
| TGME49_070880 | 1 |  | RNA binding motif-containing protein | PFD0775c |
| TGME49_008970 | 1 |  | hypothetical protein |  |
| TGME49_110570 | 1 |  | hypothetical protein |  |
| TGME49_072890 | 1 |  | RNA recognition motif domain-containing protein |  |
| TGME49_068380 | 1 |  | RNA recognition motif-containing protein |  |
| TGME49_040710 | 1 |  | RRM domain-containing protein | PF14_0028 |
| TGME49_099030 | 1 |  | RNA recognition motif 2 domain-containing protein |  |
| TGME49_113910 | 1 |  | RNA recognition motif 2 domain-containing protein |  |
| TGME49_033230 | 1 |  | RNA binding motif-containing protein | PF14_0057 |
| TGME49_113730 | 1 |  | hypothetical protein |  |
| TGME49_058610 | 1 |  | RNA recognition motif-containing protein |  |
| TGME49_024720 | 1 |  | RNA binding motif-containing zinc finger protein |  |
| TGME49_094810* | 1 |  | RRM domain-containing protein | PF10_0235 |
| TGME49_023860 | 1 |  | RNA-binding protein |  |
| TGME49_065320 | 1 |  | cell cycle control protein swf2 | PFE0750c |
| TGME49_030160 | 1 |  | conserved hypothetical protein | PF13_0058 |

*Toxoplasma* genome has 86 proteins that contain one or more RRM domains. 50 RRM proteins have orthologs in *Plasmodium falciparum*.

Data analysis performed in ToxoDB, PlasmoDB and ncbi. Conservative proteins with known function were identified in BLAST search (ncbi.org). Orthologs had e-values -10 or lower.

a – domains were identified according to ncbi.org

* - more than one *Toxoplasma* gene has the same orthologous protein in *Plasmodium falciparum*
