# Supplementary material for: Discovery of a Splicing Regulator Required for Cell Cycle Progression
Source: PLoS Genet. 2013 Feb 21;9(2):e1003305. doi: 10.1371/journal.pgen.1003305 (PMC3578776; doi:10.1371/journal.pgen.1003305)
Supplement: Table S2 — Mutation in TgRRM1 leads to global mRNA splicing defect. (DOCX) [file pgen.1003305.s011.docx]

**Table S2. Mutation in TgRRM1 leads to global mRNA splicing defect:**

| Table S2A: I/E ratios are globally increased in the mutant when grown at 40^o^C | | | |
| --- | --- | --- | --- |
| Comparison | Mean Distance  between I/E ratios | Student's T Test | p-value |
| mutant @ 34^o^C vs. mutant @ 40^o^C | 0.77 | 9.33 | 1.50E-020 |
| complemented @ 40^o^C vs. mutant @ 40^o^C | 0.69 | 8.03 | 1.15E-015 |
| mutant @ 34^o^C vs. complemented @ 40^o^C | 0.08 | 1.83 | 0.07 |

| Table S2B: I/E ratios of cell cycle regulated genes are increased in the mutant when grown at 40^o^C | | | |
| --- | --- | --- | --- |
| Comparison | Mean Distance  between I/E ratios | Student's T Test | p-value |
| G1 genes:  mutant @ 34^o^C vs. mutant @ 40^o^C | 0.70 | 11.09 | 1.97E-27 |
| G1 genes:  complemented @ 40^o^C vs. mutant @ 40^o^C | 0.51 | 2.98 | 2.94E-03 |
| G1 genes:  mutant @ 34^o^C vs. complemented @ 40^o^C | 0.19 | 1.20 | 0.23 |
| S/M genes:  mutant @ 34^o^C vs. mutant @ 40^o^C | 0.61 | 6.18 | 9.61E-10 |
| S/M genes:  complemented @ 40^o^C vs. mutant @ 40^o^C | 0.66 | 5.95 | 3.76E-09 |
| S/M genes:  mutant @ 34^o^C vs. complemented @ 40^o^C | -0.05 | -0.60 | 0.55 |
